# The effect of different exercise interventions on global cognitive function in patients with type 2 diabetes: a systematic review and network meta-analysis

**DOI:** 10.1186/s12889-026-26256-0

**Published:** 2026-01-16

**Authors:** Ming Gao, Zhiyuan Sun, Deiwei Mao, Qinghui Shang, Xuewen Tian

**Affiliations:** 1 Shandong Sport university, 10600 Century Avenue, Jinan, Shandong 250102 China; 2 Division of Physical Education, The Chinese University of Hong Kong, 2001 Longxiang Avenue, Shenzhen, 518172 China

**Keywords:** Diabetes mellitus, Type 2, Cognition, Exercise, Network Meta-Analysis

## Abstract

**Background:**

The global prevalence of type 2 diabetes mellitus (T2DM) is rising, significantly increasing the risk of cognitive impairment and dementia. Although exercise improves cognitive function in T2DM, few studies have compared different exercise modalities. This network meta-analysis assessed their effects on global cognitive function in patients with T2DM.

**Methods:**

This study systematically searched PubMed, Embase, Web of Science, Cochrane Library, China National Knowledge Infrastructure (CNKI), and Wanfang databases from their inception to October 10, 2025, and included randomized controlled trials (RCTs) evaluating the effects of exercise interventions on global cognitive function in patients with T2DM. Standardized mean differences (SMDs) and 95% confidence intervals (CIs) were pooled using a random-effects model, and treatment rankings were estimated using surface under the cumulative ranking curve (SUCRA) values. Subgroup analyses were performed according to cognitive assessment tools, intervention duration, and training frequency.

**Results:**

Twenty-one RCTs involving 2,118 participants were included. Compared with usual care, multimodal exercise (ME, 7 trials, *n* = 423, SMD = 1.04, 95% CI: 0.49–1.59), aerobic exercise (AE, 9 trials, *n* = 399, SMD = 0.85, 95% CI༚0.38–1.33), and mind–body exercise (MBE, 4 trials, *n* = 202, SMD = 0.93, 95% CI༚0.25–1.62) significantly improved global cognitive function, while resistance exercise (RE, 2 trials, *n* = 77, SMD = 0.46, 95% CI༚–0.57–1.48) showed no significant effect. SUCRA rankings indicated the highest efficacy for ME (78.1%), followed by MBE (69.0%), AE (61.5%), and RE (36.6%). Subgroup analyses showed that ME was most effective when cognition was assessed with MoCA and in long-term interventions (> 3 months), whereas MBE and AE were more effective with MMSE and in short-term interventions (≤ 3 months). At exercise frequencies ≤ 3 sessions per week, ME, AE, and MBE were effective, while at higher frequencies only ME remained effective.

**Conclusion:**

This study indicates that ME is the most effective intervention for improving global cognitive function in patients with T2DM, while MBE and AE also provide benefits. In clinical practice, exercise interventions should be tailored to individual patient characteristics to optimize cognitive outcomes.

**Supplementary Information:**

The online version contains supplementary material available at 10.1186/s12889-026-26256-0.

## Background

Type 2 diabetes mellitus (T2DM) is a common chronic metabolic disorder with a continuously increasing global prevalence [1]. It is estimated that by 2050, approximately 1.3 billion people will be affected [1]. Studies have shown that T2DM not only increases the risk of various cardiovascular and microvascular complications but also significantly elevates the likelihood of cognitive impairment and dementia [2, 3]. Relevant epidemiological studies indicate that patients with T2DM have an approximately 1.5 to 2 times higher risk of developing mild cognitive impairment (MCI) and Alzheimer’s disease [3]. Cognitive decline associated with T2DM primarily affects multiple domains, including executive function, attention, memory, and processing speed. Its underlying mechanisms may be closely related to insulin resistance, chronic hyperglycemia, neuroinflammatory responses, reduced cerebral blood perfusion, and decreased levels of brain-derived neurotrophic factor (BDNF) [4].

As there are currently no specific pharmacological interventions targeting cognitive impairment in T2DM, non-pharmacological approaches, particularly exercise interventions, are increasingly emerging as a key strategy in cognitive protection [5]. Meanwhile, the American College of Sports Medicine recommends that regular physical activity may provide psychological and cognitive benefits for patients with T2DM [6]. Most meta-analyses have demonstrated that exercise interventions can improve cognitive function in patients with T2DM. For instance, Zhang et al. reported that combined aerobic and resistance training significantly enhances global cognitive performance in this population [7]. Similarly, Lu et al. observed a pronounced intervention effect of exercise on cognitive outcomes in individuals with T2DM [8]. Previous studies have mainly focused on single exercise modalities, with limited comparisons across different types and inconsistent findings. Moreover, subgroup analyses based on cognitive assessment tools, intervention duration, or training frequency are scarce. In this study, we conducted such analyses to better interpret heterogeneity and inform individualized exercise strategies.

Network meta-analysis (NMA) is a statistical approach that simultaneously synthesizes evidence from both direct and indirect comparisons. Compared with traditional pairwise meta-analysis, NMA enables systematic head-to-head comparisons of multiple interventions within a unified analytical framework [9]. Accordingly, this study will integrate existing randomized controlled trials via NMA to compare the effects of different exercise modalities on global cognitive function in patients with T2DM, clarify the relative advantages of each intervention, and furnish more robust evidence for clinical practice and individualized treatment strategies.

## Methods

### PROSPERO

To ensure scientific rigor, methodological standardization, and full transparency, this systematic review and network meta-analysis will be conducted in strict accordance with the PRISMA 2020 statement [10], and the protocol has been prospectively registered on PROSPERO (CRD420251135559).

### Search strategy

We systematically searched PubMed, Embase, Web of Science, the Cochrane Library, CNKI, and Wanfang Data from inception to 20 October 2025. The English-database search strategy combined three core concepts: population, intervention, and outcome, integrating MeSH terms and free-text terms; all search terms were translated into Chinese for the Chinese databases. The search strategy was developed based on the PICOS framework (Population: adults with type 2 diabetes; Intervention: exercise; Comparator: any control condition; Outcomes: global cognitive function metrics; Study design: RCTs). Key search terms included: “Type 2 Diabetes” (Diabetes Mellitus, Type 2/T2DM), “Cognition” (Cognitive Function/Cognitive Dysfunction/Cognitive Impairment), “Exercise” (Physical Activity/Motor Activity). Additionally, we manually screened the reference lists of all included studies; the complete search strategies are provided in Table S1.

### Eligibility criteria

This study strictly followed the PICOS criteria for literature inclusion: (1) P (Population): patients with T2DM diagnosed according to the World Health Organization (WHO) criteria, with either normal cognitive function or MCI; [2] I (Intervention): the intervention comprised a single or multiple forms of exercise; (3) C (Comparison): the control group received usual care or no change in their original lifestyle; (4) O (Outcome): at least one quantifiable indicator of cognitive function was reported. Exclusion criteria: (1) non-RCT designs such as observational studies, case reports, or reviews; (2) populations including other diabetes types (e.g., type 1 diabetes, gestational diabetes) or individuals with severe psychiatric or neurological disorders (e.g., dementia); (3) participants with general overweight or obesity were allowed, but those requiring special treatment due to obesity-related complications were excluded; (4) studies involving only a single session of acute exercise were excluded; (5) studies with incomplete data or inability to extract required outcomes; (6) duplicate publications of identical content.

### Study selection and data extraction

Two researchers (GM and SZY) trained in evidence-based medicine independently performed study selection and data extraction. Data were extracted using a pre-designed, standardized form that captured first author, publication year, study country or region, sample size and participant characteristics (e.g., sex distribution, age range), intervention type, frequency, and duration, as well as outcome measures and associated statistical data. Discrepancies between reviewers were resolved through discussion, and, when required, a third reviewer (TXW) adjudicated to achieve consensus.

### Risk of bias assessment

The Cochrane Risk of Bias 2.0 (RoB 2) tool was employed to assess the risk of bias in the included studies [11]. Two investigators independently evaluated each trial across six domains: randomization process, deviations from the intended interventions, missing outcome data, measurement of the outcome, selection of the reported result and overall risk of bias. Each domain was judged as “low risk,” “some concerns,” or “high risk” according to the predefined criteria. Disagreements between the two reviewers were resolved through discussion with a third investigator to reach consensus.

### Statistical analysis

A network meta-analysis was performed using R 4.3.1 and Stata 14, pooling effect sizes as standardized mean differences (SMDs) with 95% confidence intervals (95% CIs). All analyses were performed under a random-effects model to better account for potential between-study heterogeneity. To further explore potential sources of heterogeneity, this study predefined several subgroup variables, including cognitive assessment scales, intervention duration, and training frequency. Regarding cognitive measures, the MoCA is more sensitive to mild cognitive impairment and executive dysfunction, whereas the MMSE is primarily used for general cognitive screening [12]; therefore, scale type was included as a stratification factor. The cutoff for intervention duration was based on prior evidence indicating that cognitive improvements typically require at least 8–12 weeks of training, and thus the duration was categorized as ≤ 12 weeks versus > 12 weeks [13, 14]. Training frequency was determined with reference to the ACSM exercise guidelines and relevant intervention studies, which commonly recommend a minimum of three sessions per week to achieve stable effects; accordingly, frequency was classified as ≤ 3 sessions/week versus > 3 sessions/week [15]. Heterogeneity was quantified with the I² statistic: values < 25% indicated low, 25%–50% moderate, 50%–75% substantial, and 75%–100% considerable heterogeneity [16]. In the presence of appreciable statistical or clinical heterogeneity, we conducted predefined subgroup and leave-one-out sensitivity analyses to delineate potential sources of heterogeneity and to evaluate the robustness of the pooled estimates.

Local inconsistency within the network was assessed using the node-splitting approach, and inconsistency was considered present when the difference between direct and indirect evidence reached statistical significance (*P* < 0.05) [17]. Furthermore, loop inconsistency tests were conducted across all closed loops, and overall inconsistency was deemed to exist when the 95% confidence interval of the inconsistency factor (IF) did not include zero [18]. To visually present the comparative efficacy among interventions, league tables and forest plots were constructed. Small-study effects and potential publication bias were initially examined through visual inspection of funnel plot symmetry, and were further evaluated using Egger’s and Begg’s tests, with *P* < 0.05 considered indicative of statistical evidence for such biases [19, 20]. The robustness of the model was verified through sensitivity analyses in which individual studies were sequentially excluded. To comprehensively appraise the relative superiority of each intervention, we calculated and reported the Surface Under the Cumulative Ranking (SUCRA) values; a higher SUCRA value indicates a more favorable rank of the intervention within the network [21]. In addition, ranking probability plots were employed to visualize the probability distributions of each intervention being the optimal choice, thereby providing a transparent basis for the formulation and hierarchical recommendation of intervention strategies in clinical practice. The literature search, study selection, analysis, and reporting procedures of this study were conducted in accordance with the PRISMA 2020 and PRISMA-NMA guidelines.

## Results

### Literature characteristics

**Fig. 1:**
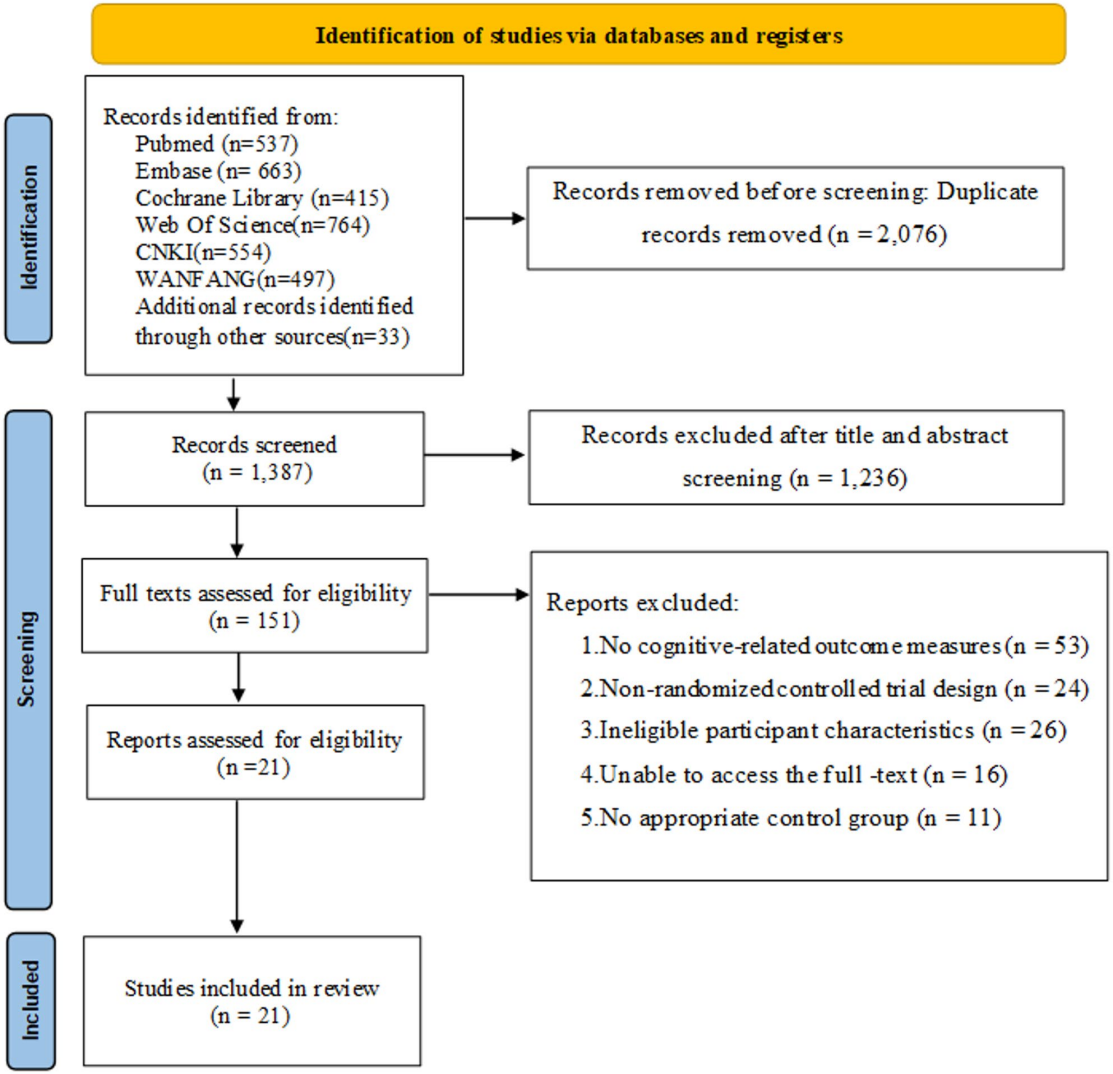
PRISMA flow diagram of literature identification and study selection

The systematic search yielded 3,463 records; after removal of duplicates, 1,387 unique citations remained. Screening of titles and abstracts excluded 1,236 studies judged irrelevant, leaving 151 articles for full-text assessment. Reasons for full-text exclusion are summarized in Table S2. Application of the predefined eligibility criteria ultimately identified 21 randomized controlled trials published between 2015 and 2024 [22–42], comprising 2,118 participants (Fig. 1). The majority of trials were conducted in Asia (*n* = 16), with China accounting for the largest proportion (*n* = 11). Interventions comprised multimodal exercise (ME), aerobic exercise (AE), resistance exercise (RE), mind–body exercise (MBE), and usual care (Usual). The specific operational procedures and representative examples for each intervention type are documented in Table S3, and the categorization was informed by ACSM’s exercise modality framework to ensure a consistent and physiologically grounded grouping [15]. The Montreal Cognitive Assessment (MoCA) was employed in 11 studies, the Mini-Mental State Examination (MMSE) in 12, and the Modified Mini-Mental State Examination (3MSE) in one. Detailed study characteristics and the complete list of included trials are presented in Table 1.

**Table 1:** The basic characteristics of the included studies

Author	Country	Numbers	Women (%)	Age	Cognition	HbA1c Mean(SD)	Intervention measures		Duration	Time (min/week)	Frequency	Outcome
							Abbreviation	Intervention content				
Chen Y 2023 [22]	China	328	50.9	60+	MCI	7.04(1.20) 6.84(1.41) 7.14(1.48)	MBE AE Usual	Tai Chi Walking Usual care	24 weeks	180	3days/wk	MOCA
Ghodrati N 2023 [23]	Iran	21	100	50–70	NA	7.10(1.20) 6.00(1.10)	ME Usual	AE + RE + Balance Usual care	12 weeks	195	3days/wk	MOCA
Ploydang T 2023 [24]	Thailand	33	63.6	60–75	MCI	7.80(0.70) 7.90(0.70)	AE Usual	Aquatic Nordic walking Usual care	12 weeks	120	3days/wk	MOCA MMSE
Wang Y 2023 [25]	China	82	50	60–75	No dementia	6.79(0.97) 7.12(1.19)	AE Usual	Outdoor aerobic training Usual care	12 months	180	3days/wk	MOCA MMSE
Yamamoto Y 2021 [26]	Japan	35	45.7	70–79	No dementia	7.40(0.90) 7.00(0.70)	RE Usual	Elastic band Usual care	48 weeks	105	Every day	MMSE
Martínez-Velilla N 2021 [27]	Spain	103	51.5	75+	No dementia	NA	ME Usual	RE + AE + Balance Usual care	8 days	≥ 200	5-7days/wk	MMSE
Molina-Sotomayor E 2021 [28]	Chile	76	100	64–78	MCI	NA	AE Usual	Walking Usual care	16 weeks	180	3days/wk	MMSE
Molina-Sotomayor E 2020 [29]	Chile	107	100	65+	MCI	8.10(0.47) 8.14(0.51)	AE Usual	Walking Usual care	6 months	180	3days/wk	MMSE
Zhu H 2015 [30]	China	78	NA	60+	MCI	NA	MBE Usual	Baduanjin Usual care	12 months	200	5days/wk	MOCA
Yanagawa M 2011 [31]	Japan	16	31.3	65–80	No dementia	7.29(0.56) 7.14(0.61)	AE Usual	Horse riding simulation equipment Usual care	12 weeks	180	4days/wk	MMSE
Cai H 2019 [32]	China	55	56.4	60–75	NA	8.90(1.97) 8.06(1.17)	MBE Usual	Qigong Usual care	12 weeks	90	3days/wk	MMSE
Matveeva MV 2019 [33]	Russia	60	65	45–60	NA	7.60(2.37) 6.90(1.04)	ME Usual	Re + Balance + Flexibility Usual care	6 months	120	2days/wk	MOCA
Zhu C 2024 [34]	China	108	48.1	45+	No dementia	NA	RE Usual	Resistance exercise Usual care	2 weeks	NA	3-5days/wk	MOCA
Zhang J 2017 [35]	China	74	54.1	60+	MCI	7.92(1.05) 8.45(1.63)	AE Usual	Walking Usual care	3 months	≥ 120	Every day	MOCA
Liang C 2017 [36]	China	100	48	60+	MCI	8.26(1.19) 8.31(1.23)	AE Usual	Aerobic exercises Usual care	3 months	≥ 240	Every day	MMSE
Wei Z 2003 [37]	China	120	52.5	65+	MCI	8.26(1.19) 8.31(1.23)	AE Usual	Walking + gymnastics Usual care	3 months	200	3days/wk	MOCA
Liu J 2023 [38]	China	128	46.1	37–77	NA	8.30(0.23) 8.32(0.28)	ME Usual	AE + RE Usual care	3 months	300	Every day	MOCA
Huang Z 2022 [39]	China	96	41.7	45+	NA	NA	ME Usual	AE + Tai chi, Baduanjin Usual care	3 months	300	3-5days/wk	MMSE
Wang J 2015 [40]	China	87	NA	60–70	NA	8.39(1.92) 8.37(1.33)	MBE Usual	Baduanjin Usual care	12 months	180	3days/wk	MOCA MMSE
Espeland MA 2017 [41]	USA	415	67.7	70–89	NA	NA	ME Usual	Walking, strength, and balance Usual care	2 years	≥ 150	3-4days/wk	3MSE
Ghahfarrokhi MM 2024 [42]	Iran	32	NA	60+	MCI	8.88(1.91) 8.85(1.85)	ME Usual	Endurance, strength, balance Usual care	6 weeks	90	3days/wk	MMSE

*ME* Multimodal exercise, *MBE* Mind–body exercise, *RE* Resistance exercise, *AE* Aerobic exercise, *Usual* usual care, *wk* week, *NA* Not Available, *MCI* Mild cognitive impairment

### Quality evaluation

**Fig. 2:**
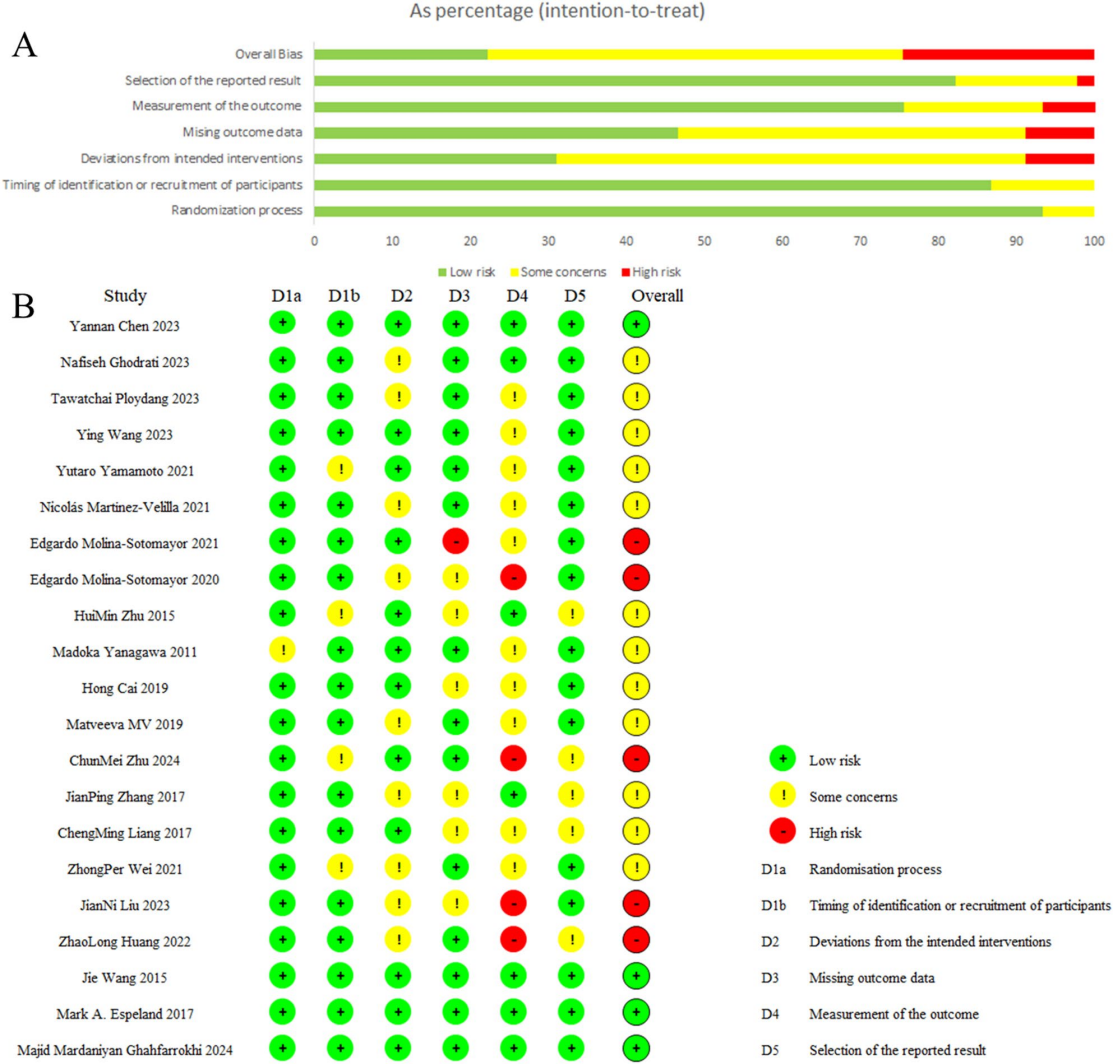
Risk-of-bias summary for the 21 randomized controlled trials assessed with the Cochrane RoB 2 tool. **A** Proportion of trials assigned low, some-concern, or high risk for each domain, expressed as percentages. **B** Individual risk-of-bias judgments for all six domains across each of the 21 included trials

Application of the Cochrane RoB 2.0 tool indicated that, across the 21 included trials (Fig. 2), 7 were rated as having a high risk of bias, 12 as raising some concerns, and 2 as exhibiting a low risk of bias. The distribution of risk judgments across the six RoB 2 domains and the overall assessment is presented as the percentage of trials rated low, some concerns, or high risk: Randomization process (90.5%, 9.5%, 0%); Deviations from intended interventions (42.9%, 57.1%, 0%); Missing outcome data (52.4%, 28.6%, 19.0%); Measurement of the outcome (28.6%, 47.6%, 23.8%); Selection of the reported result (81.0%, 19.0%, 0%); and overall bias (9.5%, 57.1%, 33.3%).

### Network meta-analysis

#### Network evidence plot

**Fig. 3:**
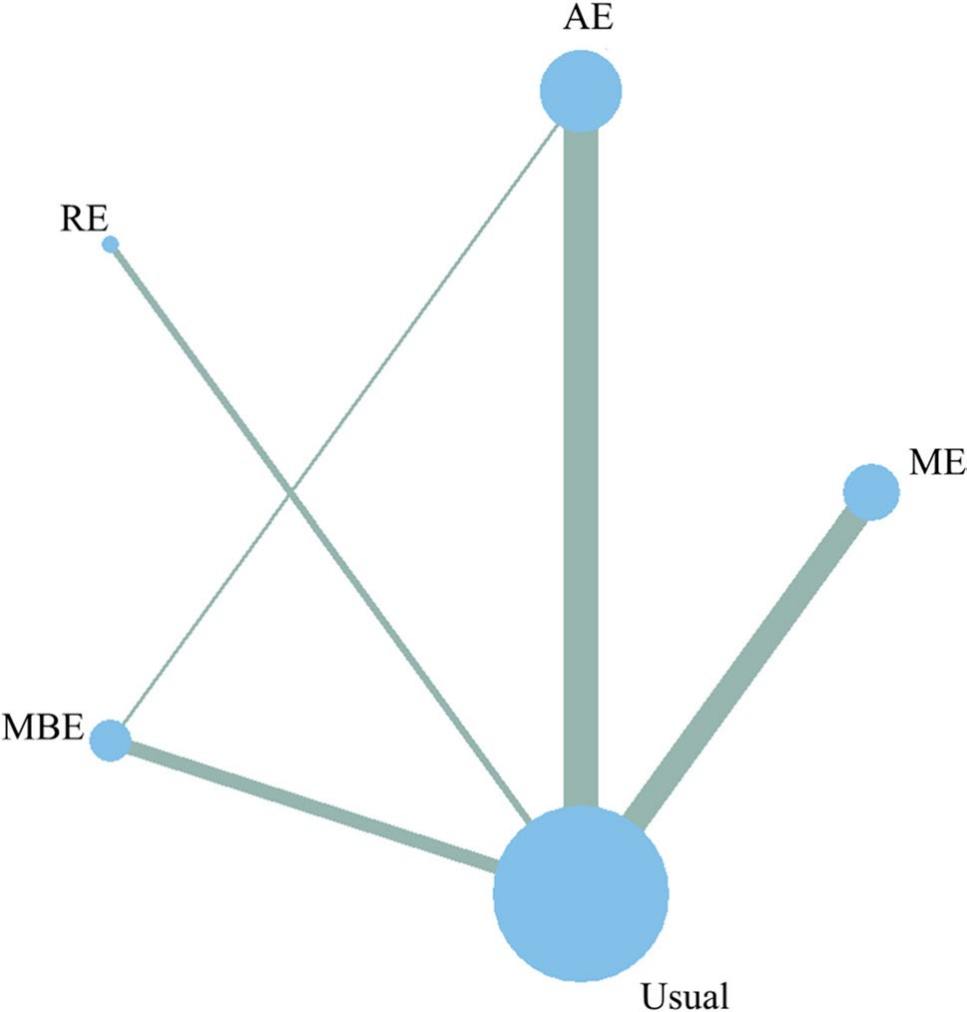
Network geometry illustrating direct and indirect comparisons among the included interventions

Figure 3 depicts the network of direct comparisons between each exercise intervention and the control group. Nodes represent interventions, with their diameters proportional to the number of participants assigned to each modality; connecting lines denote direct pairwise comparisons, and their thickness is proportional to the number of trials contributing to each comparison.

### Combined effect sizes

**Fig. 4:**
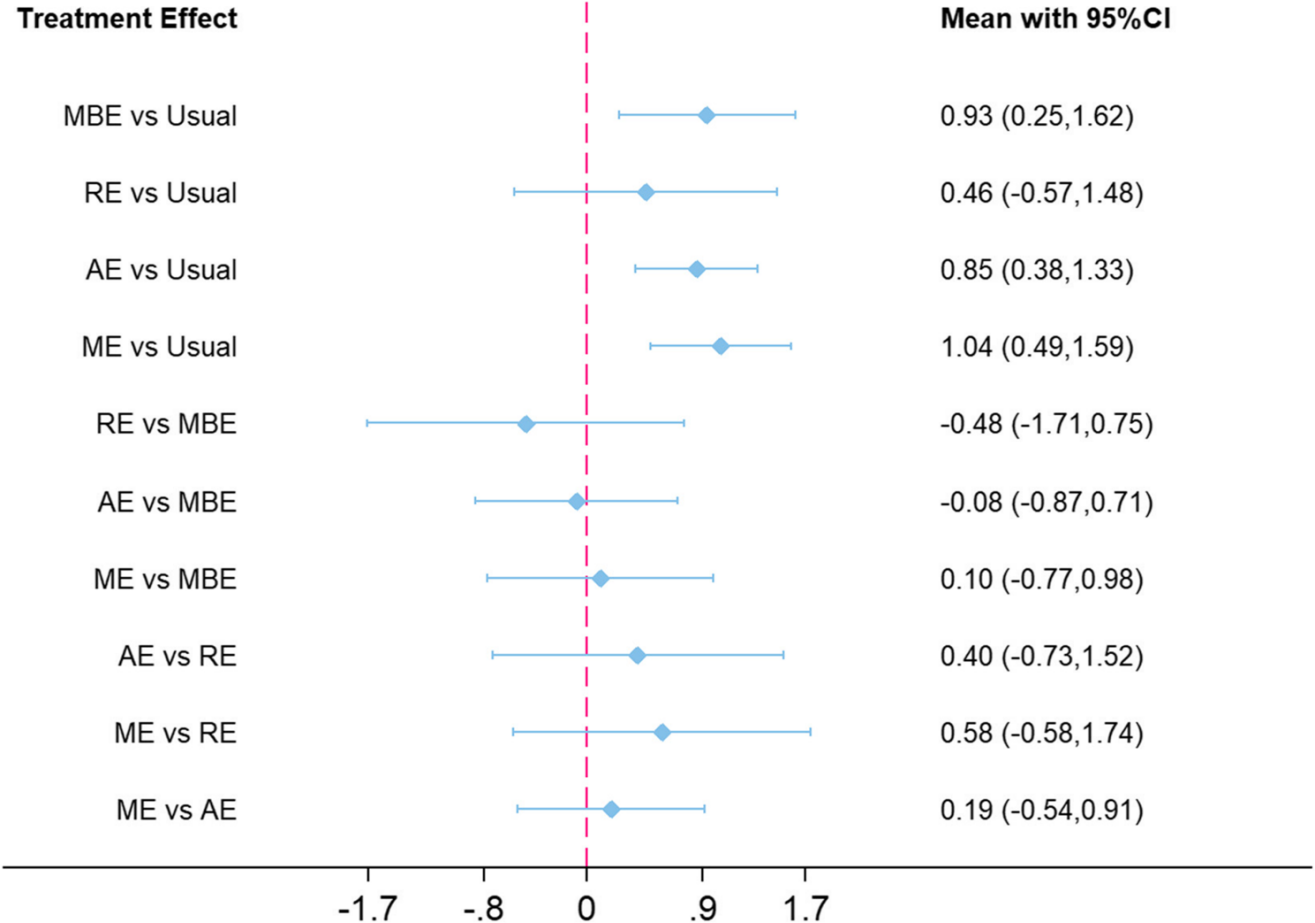
Forest plot of the network meta-analysis depicting standardized mean differences (SMDs) with 95% confidence intervals for pairwise comparisons among all interventions, illustrating both the magnitude and direction of treatment effects

Network meta-analysis revealed that, relative to Usual, ME (SMD = 1.04, 95% CI: 0.49–1.59), AE (SMD = 0.85, 95% CI: 0.38–1.33), and MBE (SMD = 0.93, 95% CI: 0.25–1.62) all exerted statistically significant benefits on global cognitive function. Although the effect estimate for RE (SMD = 0.46, 95% CI: − 0.57 to 1.48) did not reach conventional statistical significance, the point estimate still favored RE over Usual. The comparative efficacy among interventions is visually presented in the league table and forest plot (Fig. 4; Table 2).

**Table 2:** League table presenting the pooled standardized mean differences (SMDs) with 95% confidence intervals for all head-to-head comparisons derived from the network meta-analysis

ME				
0.19(−0.54,0.91)	AE			
0.58(−0.58,1.74)	0.40(−0.73,1.52)	RE		
0.10(−0.77,0.98)	−0.08(−0.87,0.71)	−0.48(−1.71,0.75)	MBE	
**1.04(0.49,1.59)**	**0.85(0.38**,**1.33)**	0.46(−0.57,1.48)	**0.93(0.25**,**1.62)**	Usual

Values in bold indicate statistically significant effects, where the 95% credible interval does not include 0

### SUCRA rankings and ranking probability

**Fig. 5:**
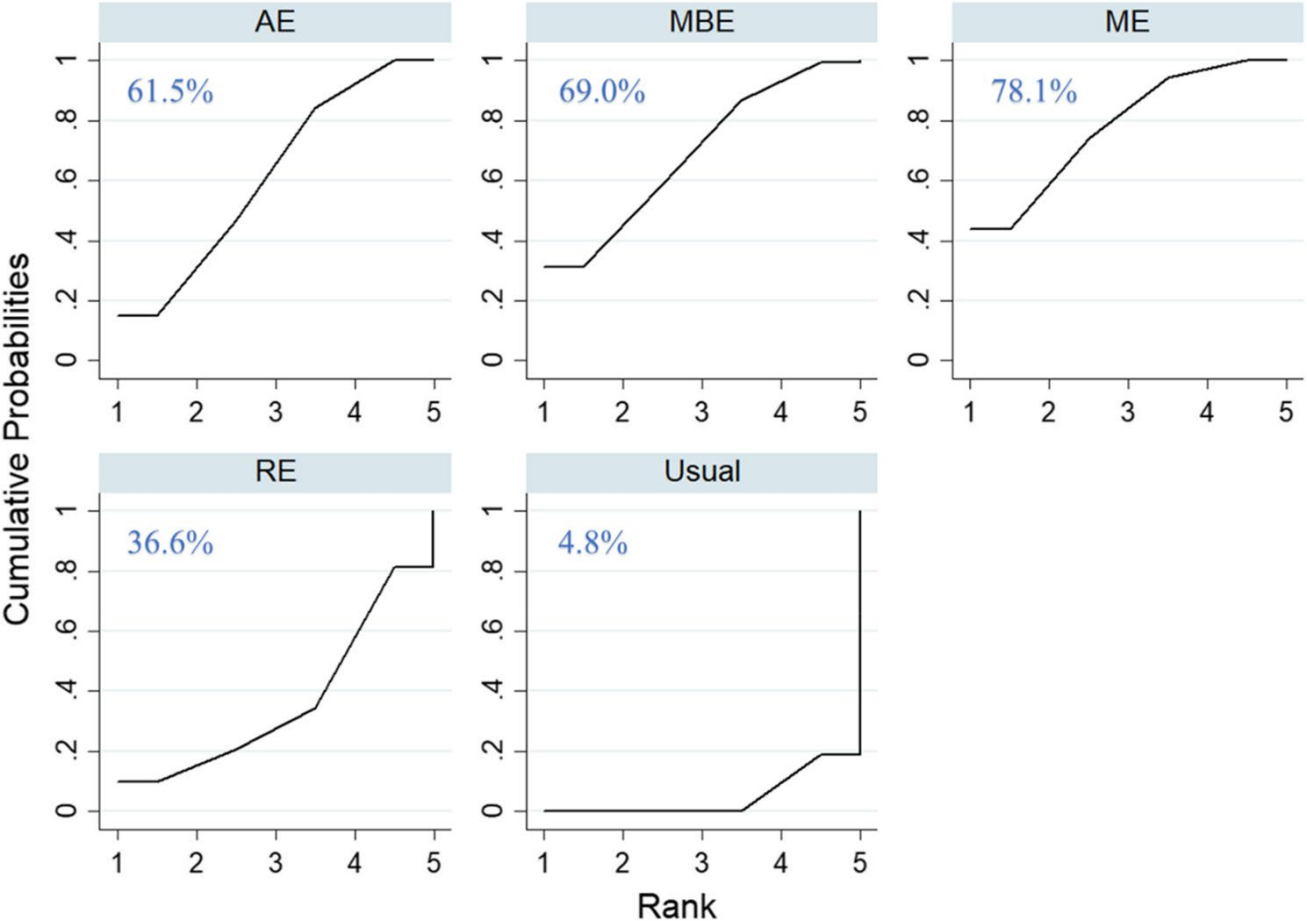
Comparative efficacy of interventions based on the Surface Under the Cumulative Ranking curve, where higher SUCRA values denote superior treatment effects

According to the SUCRA-based hierarchical analysis (Fig. 5), ME attained the highest SUCRA value (78.1%) and therefore ranked as the most effective intervention for improving global cognitive function in this network. This was followed sequentially by MBE (69.0%), AE (61.5%), and RE (36.6%). Ranking probability plots were additionally constructed to graphically quantify the posterior probabilities of each intervention’s relative efficacy, thereby elucidating the hierarchical certainty of the evidence (Figure S1).

### Heterogeneity and model validation

**Fig. 6:**
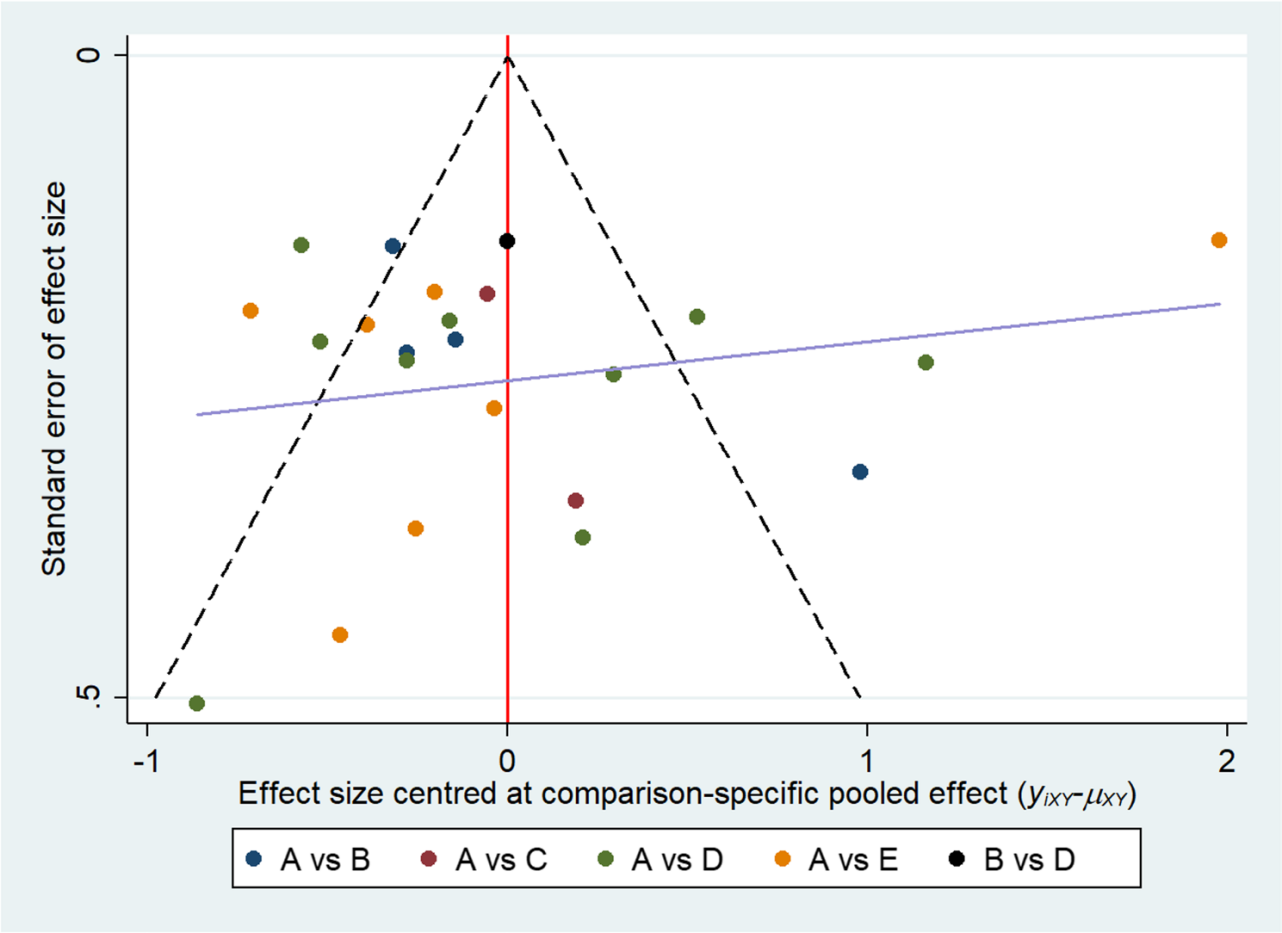
Relative corrected funnel plots for publication bias: (**A**) Usual; (**B**) MBE; (**C**) RE; (**D**) AE; (**E**) ME

Heterogeneity and model consistency were assessed using consistency checks and the node-splitting approach. The random-effects model revealed considerable between-study heterogeneity (I² = 93.3%). Overall consistency testing showed that the network was consistent (*P* = 0.686), and node-splitting analysis revealed no significant discrepancies between direct and indirect evidence (*P* > 0.05), indicating good network consistency among the included studies. Assessment of publication bias showed that neither Begg’s test (z = 1.33, *P* = 0.185) nor Egger’s test (t = −0.71, *P* = 0.484) indicated significant bias. The corrected funnel plot (Fig. 6) was largely symmetrical; despite a few elongated effect lines, the overall distribution was balanced, suggesting that the findings of this network meta-analysis are robust.

### Subgroup analyses

To elucidate potential sources of heterogeneity, we conducted a priori subgroup analyses stratified by assessment instrument, intervention duration, and training frequency.

### Subgroup analysis by cognitive assessment instrument

**Fig. 7:**
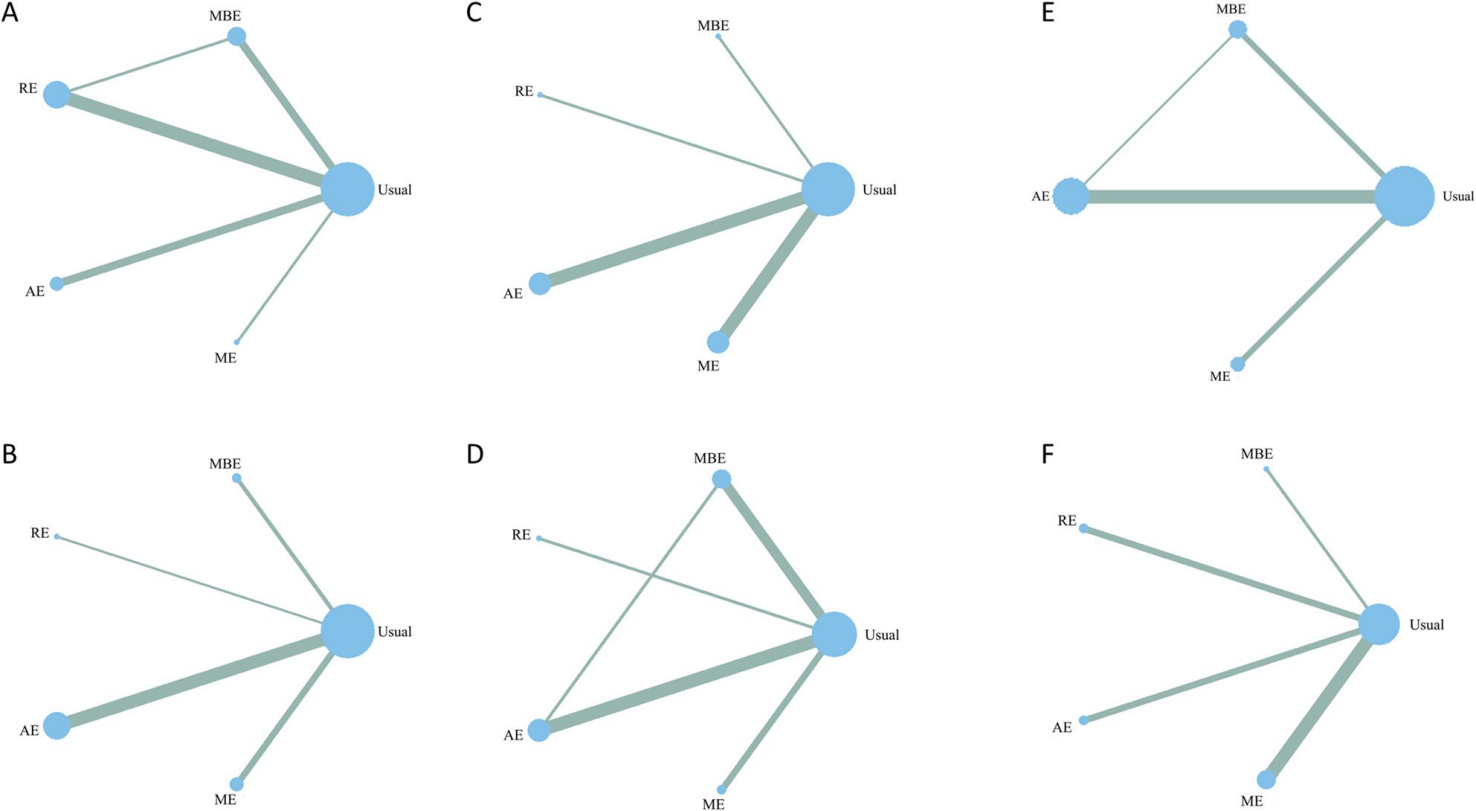
Network plots for subgroup analyses: (**A**) MoCA; (**B**)MMSE; (**C**) short-term intervention (≤ 3 months); (**D**) long-term intervention (> 3 months); (**E**) training frequency ≤ 3 days per week; (**F**) training frequency > 3 days per week

According to the cognitive assessment tools used, the included studies were divided into the MoCA group and the MMSE group for subgroup analysis. In the MoCA group, the corresponding network plot, forest plot, and league table are shown in Fig. 7A, Figure S2, and Table S4, respectively. Compared with Usual, ME (SMD = 0.82, 95% CI: 0.32–1.32), AE (SMD = 0.77, 95% CI: 0.42–1.13), and MBE (SMD = 0.71, 95% CI: 0.29–1.13) exhibited significant beneficial effects, while other interventions showed no statistically significant differences. In the MMSE group, the corresponding network plot, forest plot, and league table are shown in Fig. 7B, Figure S3, and Table S5, respectively. The results showed that AE (SMD = 0.85, 95% CI: 0.35–1.36) and MBE (SMD = 1.18, 95% CI: 0.32–2.04) significantly improved cognitive function, whereas ME (SMD = 0.58, 95% CI: −0.14–1.31) demonstrated a positive trend but did not reach statistical significance, as its confidence interval included zero. Since only a single study employed the 3MSE scale, there was insufficient data to perform a subgroup analysis, and it was thus excluded from the group comparisons.

### Subgroup analysis according to the duration of the intervention

The included studies were categorized into short-term (≤ 3 months) and long-term (> 3 months) groups according to intervention duration. In the short-term intervention group, the results showed that, compared with Usual, ME (SMD = 0.60, 95% CI: 0.29–0.92), AE (SMD = 0.83, 95% CI: 0.50–1.16), and MBE (SMD = 1.82, 95% CI: 1.02–2.62) all demonstrated significant intervention effects, with MBE exhibiting a higher effect size than the other interventions, the network plot is shown in Fig. 7C, and the corresponding forest plot and league table are presented in Figure S4 and Table S6. In the long-term intervention group, ME (SMD = 2.03, 95% CI: 0.89–3.17) and AE (SMD = 0.91, 95% CI: 0.12–1.69) continued to show significant improvements, whereas MBE, although exhibiting a positive trend, did not reach statistical significance as its confidence interval included zero. Other interventions did not demonstrate significant effects in the long-term group, the intervention comparisons and network plot are shown in Fig. 7D, with the corresponding forest plot and league table presented in Figure S5 and Table S7.

### Subgroup analysis according to training frequency

To investigate the potential influence of training frequency on intervention outcomes, the included studies were categorized into two groups according to weekly training frequency: ≤3 days per week and > 3 days per week. Among studies with a training frequency of ≤ 3 days per week, ME (SMD = 0.79, 95% CI: 0.08–1.94), AE (SMD = 0.87, 95% CI: 0.46–1.28), and MBE (SMD = 1.03, 95% CI: 0.43–1.64) significantly improved cognitive function compared with Usual. No studies on RE were available for this frequency group, the relevant network diagram, forest plot, and league table are presented in Fig. 7E, Figure S6, and Table S8, respectively. Among studies with a training frequency of > 3 days per week, only ME (SMD = 1.19, 95% CI: 0.16–2.22) exhibited a significant effect, whereas other interventions did not show statistically significant differences. The intervention comparisons, network plot, forest plot, and league table are presented in Fig. 7F, Figure S7, and Table S9, respectively.

### Sensitivity analysis

A sensitivity analysis was performed on the remaining 14 studies after excluding seven studies assessed as having a high risk of bias. The findings indicated that the primary effect sizes were stable and aligned with the results of the main analysis. Compared with Usual, ME (SMD = 1.30, 95% CI: 0.51–2.08), AE (SMD = 0.66, 95% CI: 0.02–1.29), and MBE (SMD = 0.91, 95% CI: 0.15–1.66) maintained significant effects, while RE (SMD = 0.34, 95% CI: −1.18–1.87) remained non-significant. The related forest plot and league table are presented in Figure S8 and Table S10.

## Discussion

Based on a network meta-analysis, this study systematically evaluated the effects of various exercise interventions on global cognitive function in patients with T2DM. The results indicated that ME demonstrated the most significant benefits in improving cognitive function, followed by MBE and AE. In contrast, although RE showed a positive trend, its effects did not reach statistical significance. Further sensitivity analyses confirmed the robustness and consistency of the main findings. To our knowledge, this is the first network meta-analysis focusing on the impact of exercise interventions on global cognitive function in T2DM patients, offering evidence-based guidance regarding the relative efficacy of different exercise modalities.

Emerging evidence suggests that exercise may ameliorate cognitive function in patients with T2DM through multiple mechanisms. Firstly, physical activity enhances insulin sensitivity and optimizes cerebral glucose metabolism, thereby improving cognitive performance [43]. Cerebral insulin resistance, commonly observed in T2DM patients, is closely linked to cognitive decline. A study by Kullmann et al. [44] demonstrated that an 8-week exercise intervention restored brain insulin action in sedentary adults, accompanied by improved cerebral perfusion and functional connectivity within neural networks—particularly in the striatal region. Moreover, exercise modulates signaling pathways such as AMPK/SIRT1 and PI3K/Akt, which promote neuroplasticity and upregulate the expression of neurotrophic factors, ultimately enhancing synaptic plasticity and cognitive function [45]. Additionally, physical activity reduces levels of inflammatory markers and mitigates neuroinflammation, thereby preserving brain structure and function [46]. Animal studies have further corroborated that exercise increases the expression of BDNF, improves cerebral blood flow, and supports neuronal growth and repair, all contributing to enhanced cognition. Notably, recent research indicates that exercise may also exert its benefits via modulation of the gut microbiota [47]. AE has been shown to improve cognitive function in T2DM mice, partly through alterations in gut microbiome composition [48], suggesting a potential role of the gut–brain axis in mediating exercise-induced cognitive benefits. The primary findings of this study align closely with the previously described mechanisms and further advance the theoretical framework supporting exercise interventions for the enhancement of cognitive function in individuals with T2DM. ME, as a combined intervention incorporating two or more types of exercise, may confer benefits to brain health through distinct signaling pathways, thereby producing synergistic gains across multiple cognitive domains [49]. Sun et al. [50] demonstrated that ME can effectively attenuate diabetes-related cognitive decline by enhancing neural plasticity and modulating multiple insulin- and inflammation-related signaling pathways. Similarly, Zhang et al. [7] confirmed that ME activates prefrontal cortex activity and improves brain network efficiency, leading to comprehensive improvements in executive function, memory, and other cognitive domains, thereby further supporting the advantages of ME observed in the present study. This study found that AE also significantly improves global cognitive function, consistent with the findings of Ding et al. [51]. AE may indirectly enhance cognitive function by increasing energy expenditure, improving lipid metabolism, reducing body fat, increasing lean mass, enhancing immune function, and improving insulin sensitivity. Notably, there is currently a lack of systematic reviews or meta-analyses specifically examining the effects of MBE on global cognitive function in patients with T2DM. Some studies suggest that MBE may support cognitive processing by enhancing autonomic nervous system function and regulating the balance between sympathetic and parasympathetic activity, thereby improving cardiovascular function and cerebral perfusion [52]. Additionally, it may enhance cognitive function by promoting intrinsic hippocampal activity, increasing hippocampal volume, and normalizing aberrant hyperactivity within the hippocampus [53]. Although RE did not reach statistical significance in the present study, its direction of effect is consistent with previously reported potential cognitive benefits [50]. Existing evidence suggests that RE may confer cognitive advantages by promoting the secretion of neurotrophic factors, improving cerebral hemodynamics, and modulating neuroinflammation [54, 55]. The limited number of studies on RE and the resulting sparse network nodes may increase the uncertainty of effect estimates, warranting cautious interpretation of the RE-related findings. Overall, the prominent effects of ME, together with the positive impacts observed for AE and MBE, collectively support the potential of exercise interventions to improve global cognitive function in individuals with T2DM and underscore the importance of ME as an optimized strategy.

The subgroup analyses in this study indicate that the choice of cognitive assessment tools, intervention duration, and training frequency may influence the effects of exercise interventions on cognitive improvement in patients with T2DM.

Previous studies have indicated that MoCA exhibits a sensitivity of 90% and specificity of 87% in detecting MCI, whereas the MMSE has a sensitivity of only 18%, despite a specificity of 100% [56, 57]. In differentiating Alzheimer’s disease and MCI, MoCA demonstrates superior discriminative ability and classification accuracy for MCI compared to the MMSE [58]. This discrepancy may stem from differences in sensitivity and cognitive domain coverage between the two instruments: MoCA is more effective at detecting improvements in higher-order cognitive domains such as executive function, attention, and abstract thinking [59], while the MMSE primarily assesses basic cognitive functions like orientation, memory, and language. The limited sensitivity of the MMSE to mild or early cognitive changes may lead to an underestimation of interventional benefits [60]. Overall, the selection of cognitive assessment instruments may materially shape the estimated effects of exercise interventions, underscoring the need for rigorous and context-appropriate tool selection in future studies to mitigate bias and more accurately characterize intervention efficacy.

Based on the duration of intervention, the included studies were stratified into a short-term (≤ 3 months) group and a long-term (> 3 months) group. The results revealed that in the short-term intervention group, MBE yielded relatively high effect sizes, potentially attributable to its beneficial effects on resting-state functional connectivity, autonomic nervous regulation, and emotional management, which may indirectly improve executive function and attention [53, 61]. However, its advantage tended to diminish with extended intervention periods, suggesting that the duration and intensity of MBE protocols require further optimization. AE demonstrated consistent benefits across both short- and long-term interventions. Regular AE has been shown to enhance cognitive performance by improving cardiorespiratory fitness, increasing insulin sensitivity, and promoting cerebral blood flow and oxygenation—a mechanism repeatedly corroborated by neuroimaging and animal studies [62–64]. ME consistently conferred cognitive benefits across varying intervention durations, potentially due to its multimodal composition that engages complementary physiological and neurocognitive mechanisms. Consequently, it yielded more substantial and sustainable cognitive improvements in long-term interventions [49]. It is noteworthy that RE did not demonstrate statistically significant benefits in either short- or long-term interventions, suggesting that it may be insufficient alone to improve global cognitive function in patients with T2DM.

The results indicated that at a frequency of ≤ 3 sessions per week, ME, AE, and MBE all significantly improved global cognitive function in patients with T2DM. In contrast, in the high-frequency group (> 3 sessions/week), only ME maintained statistically significant benefits, while no significant effects were observed for the other exercise modalities. The frequency-based subgroup analysis indicates that intervention efficacy is jointly shaped by training frequency and intervention duration. ME demonstrated consistent benefits across different training frequencies, likely owing to its integrated structure combining aerobic, resistance, and mind-body components. This comprehensive approach simultaneously targets multiple mechanisms, including improved insulin sensitivity, enhanced cerebral blood flow, upregulation of neurotrophic factors (e.g., BDNF), and modulation of emotional and cognitive networks [65, 66]. For interventions delivered at lower training frequencies (≤ 3 sessions/week), prior research indicates that such frequencies are sufficient to stimulate the secretion of neurotrophic factors, thereby promoting synaptic plasticity and neural network remodeling [67]. However, at higher training frequencies (> 3 sessions/week), intervention efficacy may depend more heavily on the sufficient accumulation of duration and overall training load. If high-frequency training is of short duration or insufficient intensity, the underlying neurophysiological and psychological mechanisms may not be fully activated, which could explain why some studies fail to show significant effects [68, 69]. Moreover, existing evidence suggests a positive overall association between exercise dose and cognitive health in older adults [70], indicating that the combined effect of frequency, duration, and intensity may be a key determinant of intervention efficacy. These findings should be interpreted with caution, particularly given that intervention effects may differ across varying combinations of frequency and duration.

## Conclusion

This study demonstrates that multiple types of exercise interventions can improve global cognitive function in patients with T2DM, among which ME yielded the most significant benefits, particularly in long-term interventions. MBE and AE also showed positive effects, though the observed outcomes were influenced by intervention duration and the choice of cognitive assessment tools. These findings support the integration of exercise interventions as a key strategy for managing cognitive function in T2DM patients. Future research should further elucidate the underlying mechanisms and refine intervention protocols to enable more precise and individualized approaches.

### Limitations

Despite strict adherence to the PRISMA 2020 guidelines for literature screening and data extraction, this study is subject to several limitations. First, several included studies were at risk of bias due to lack of blinding and incomplete outcome data, potentially affecting the reliability of effect estimates. Second, the predominance of study populations from Asia, especially China, may constrain the generalizability of the findings. Third, heterogeneity arising from differences in intervention protocols, participant characteristics, and outcome assessments underscores the need for more standardized designs in future studies to improve evidence robustness and comparability. Finally, while SUCRA rankings provide a hierarchy of intervention effects, interpretation should account for clinical feasibility and patient adherence.

Based on these limitations, future research should prioritize the following directions. First, multicenter, large sample RCTs are needed to strengthen evidence robustness and generalizability. Second, differential responses in cognitively at-risk populations should be investigated to inform individualized exercise prescriptions. Third, exercise dose should be optimized, with standardized intensity and monitoring of participant adherence to ensure accurate assessment of intervention effects. Fourth, integration of neuroimaging and biomarker assessments may further clarify the mechanisms underlying exercise-induced cognitive improvements. Overall, these findings provide actionable guidance for selecting ME and well-designed interventions, supporting individualized, evidence-based strategies to enhance cognitive function in individuals with T2DM.

## Supplementary Information

Supplementary Material 1.

## Data Availability

All data generated or analyzed during this study are included in this published article and supplementary materials.

## Abbreviations

T2DM: Type 2 Diabetes Mellitus
NMA: Network Meta-Analysis
ME: Multimodal exercise
MBE: Mind-body exercise
AE: Aerobic exercise
RE: Resistance exercise
Usual: Usual care
SUCRA: Surface Under the Cumulative Ranking Curve
MCI: Mild Cognitive Impairment
BDNF: Brain-Derived Neurotrophic Factor
MOCA: Montreal Cognitive Assessment
MMSE: Mini-Mental State Examination
3MSE: Modified Mini-Mental State Examination
