# Supplementary material for: The effect of different exercise interventions on global cognitive function in patients with type 2 diabetes: a systematic review and network meta-analysis
Source: BMC Public Health. 2026 Jan 16;26:559. doi: 10.1186/s12889-026-26256-0 (PMC12892726; doi:10.1186/s12889-026-26256-0)
Supplement: Supplementary file 1 — Supplementary Material 1. [file 12889_2026_26256_MOESM1_ESM.docx]

**Table S1 Literature search strategy.**

1. **PubMed**

| Search | Query |
| --- | --- |
| #1 | "Diabetes Mellitus, Type 2"[Mesh] OR "Type 2 Diabetes Mellitus"[tiab] OR "T2DM"[tiab] |
| #2 | "Cognition"[Mesh] OR "Cognitive Dysfunction"[Mesh] OR "Cognitive Impairment"[tiab] OR "Cognitive Function"[tiab] OR "Neurocognitive"[tiab] |
| #3 | "Exercise"[Mesh] OR "Motor Activity"[Mesh] OR "Exercise Therapy"[Mesh] OR "Physical Activity"[tiab] OR "Physical Training"[tiab] OR "Movement Intervention"[tiab] OR "Mind-Body Therapies"[Mesh] |
| #4 | randomized controlled trial[pt] OR controlled clinical trial[pt] OR randomized[tiab] OR placebo[tiab] OR "clinical trial"[tiab] OR "RCT"[tiab] |
| #5 | #1 AND #2 AND #3 AND #4 |

**2. Cochrane Library**

| Search | Query |
| --- | --- |
| #1 | [mh "Diabetes Mellitus, Type 2"] OR "type 2 diabetes" OR "T2DM" OR "type II diabetes" OR "adult-onset diabetes" OR "non-insulin dependent diabetes" |
| #2 | [mh "Cognition"] OR [mh "Cognitive Dysfunction"] OR "cognitive function" OR "cognitive impairment" OR "cognitive decline" OR "cognitive dysfunction" OR "executive function" OR "neurocognitive" OR "memory impairment" |
| #3 | [mh "Exercise Therapy"] OR [mh "Motor Activity"] OR [mh "Physical Fitness"] OR "exercise" OR "physical activity" OR "physical exercise" |
| #4 | "randomized controlled trial" OR "randomised controlled trial" OR "RCT" OR "clinical trial" OR "intervention study" |
| #5 | #1 AND #2 AND #3 AND #4 |

**3. Embase**

| Search | Query |
| --- | --- |
| #1 | 'type 2 diabetes mellitus'/exp OR 'type 2 diabetes':ti,ab OR 'T2DM':ti,ab OR 'non-insulin dependent diabetes':ti,ab OR 'adult-onset diabetes':ti,ab |
| #2 | 'cognitive defect'/exp OR 'cognitive dysfunction'/exp OR 'cognition'/exp OR 'cognitive impairment':ti,ab OR 'cognitive function':ti,ab OR 'neurocognitive':ti,ab OR 'memory loss':ti,ab OR 'executive function':ti,ab |
| #3 | 'exercise'/exp OR 'motor activity'/exp OR 'physical activity':ti,ab OR 'exercise therapy':ti,ab OR 'mind body exercise':ti,ab |
| #4 | 'randomized controlled trial'/exp OR 'randomization'/exp OR 'placebo'/exp OR randomized:ti,ab OR 'randomised':ti,ab OR 'rct':ti,ab OR 'clinical trial':ti,ab OR 'intervention study':ti,ab |
| #5 | #1 AND #2 AND #3 AND #4 |

**4. Web of Science**

| Search | Query |
| --- | --- |
| #1 | TS=("type 2 diabetes" OR "T2DM" OR "type II diabetes" OR "adult-onset diabetes" OR "non-insulin dependent diabetes") |
| #2 | TS=("cognition" OR "cognitive function" OR "cognitive impairment" OR "cognitive decline" OR "cognitive dysfunction" OR "neurocognition" OR "executive function" OR "memory impairment") |
| #3 | TS=("exercise" OR "physical activity" OR "physical exercise" OR "exercise therapy" OR "motor activity" OR "movement intervention") |
| #4 | TS=("randomized controlled trial" OR "randomised controlled trial" OR "RCT" OR "clinical trial" OR "intervention study") |
| #5 | #1 AND #2 AND #3 AND #4 |

**5. CNKI**

| Search | Query |
| --- | --- |
| #1 | (ab,kw,ti=diabetes+type 2 diabetes+T2DM+adult-onset diabetes+non-insulin dependent diabetes) AND (ab,kw,ti=elderly+older adults+older people+aging population+senior citizens+older individuals) AND (ab,kw,ti=cognition+cognitive function+cognitive impairment+mild cognitive impairment+cognitive decline+neurocognitive+mental behavior+executive function+memory loss) AND (ab,kw,ti=exercise+physical activity+exercise training+exercise) |

**6. WanFang**

| Search | Query |
| --- | --- |
| #1 | (ab,kw,ti=diabetes+type 2 diabetes+T2DM+adult-onset diabetes+non-insulin dependent diabetes) AND (ab,kw,ti=elderly+older adults+older people+aging population+senior citizens+older individuals) AND (ab,kw,ti=cognition+cognitive function+cognitive impairment+mild cognitive impairment+cognitive decline+neurocognitive+mental behavior+executive function+memory loss) AND (ab,kw,ti=exercise+physical activity+exercise training+exercise) |

**Table S2** List of excluded studies

| **Year** | **Title** | **DOI** | **Reason for exclusion** |
| --- | --- | --- | --- |
| 2022 | Effectiveness of a Community-Based Structured Physical Activity Program for Adults With Type 2 Diabetes: A Randomized Clinical Trial. | https://doi.org/10.1001/jamanetworkopen.2022.47858 | No cognitive outcomes: not reported |
| 2019 | Effect of a Behavioral Intervention Strategy on Sustained Change in Physical Activity and Sedentary Behavior in Patients With Type 2 Diabetes: The IDES_2 Randomized Clinical Trial. | https://doi.org/10.1001/jama.2019.0922 | No cognitive outcomes: not reported |
| 2021 | A randomized controlled trial of a structured program combining aerobic and resistance exercise for adults with type 2 diabetes in Japan | https://doi.org/10.1007/s13340-021-00506-5 | No cognitive outcomes: not reported |
| 2020 | The Effect of a Novel Low-Volume Aerobic Exercise Intervention on Liver Fat in Type 2 Diabetes: A Randomized Controlled Trial. | https://doi.org/10.2337/dc20-0403 | No cognitive outcomes: not reported |
| 2023 | The effect of home-based resistance exercise training in people with type 2 diabetes: A randomized controlled trial | https://doi.org/10.1002/dmrr.3677 | No cognitive outcomes: not reported |
| 2024 | Effectiveness of structured exercise program on insulin resistance and functional capacity in adults with type 2 diabetes: a randomized trial. | https://doi.org/10.1371/journal.pone.0302831 | No cognitive outcomes: not reported |
| 2022 | Technology-based and supervised exercise interventions for people with type 2 diabetes: a randomized trial. | https://doi.org/10.1016/j.jneb.2021.09.001 | No cognitive outcomes: not reported |
| 2022 | Effects of Exercise Intervention on Type 2 Diabetes: A 16-week Combined Aerobic and Resistance Training Trial. | https://doi.org/10.3389/fendo.2022.937264 | No cognitive outcomes: not reported |
| 2017 | Effect of an Intensive Lifestyle Intervention on Glycemic Control in Patients With Type 2 Diabetes: A Randomized Clinical Trial | https://doi.org/10.1001/jama.2017.10169 | No cognitive outcomes: not reported |
| 2021 | Physical-cognitive training effects on older adults with type 2 diabetes: group-based intervention study. | https://doi.org/10.12965/jer.2142106.053 | No cognitive outcomes: not reported |
| 2018 | Evaluating the clinical implementation of structured exercise for people with type 2 diabetes: randomized pragmatic trial. | https://doi.org/10.1186/1471-2458-11-655 | No cognitive outcomes: not reported |
| 2022 | The effects of aerobic exercise combined with resistance training on inflammatory factors and heart rate variability in middle-aged and elderly women with type 2 diabetes mellitus | https://doi.org/10.1111/anec.12996 | No cognitive outcomes: not reported |
| 2022 | Effects of 12-Week Progressive Sandbag Exercise Training on Glycemic Control and Muscle Strength in Patients with Type 2 Diabetes Mellitus Combined with Possible Sarcopenia | https://doi.org/10.3390/ijerph192215009 | No cognitive outcomes: not reported |
| 2010 | Impact of diverse aerobic exercise plans on glycemic control, lipid levels, and functional activity in stroke patients with type 2 diabetes mellitus | https://doi.org/10.3389/fendo.2024.1389538 | No cognitive outcomes: not reported |
| 2024 | Exploring the Feasibility of Digital Voice Assistants for Delivery of a Home-Based Exercise Intervention in Older Adults With Obesity and Type 2 Diabetes Mellitus: Randomized Controlled Trial | https://preprints.jmir.org/preprint/53064 | No cognitive outcomes: not reported |
| 2025 | Digital outdoor exercise program for obese patients with type 2 diabetes mellitus: a non-inferiority randomized controlled trial | https://doi.org/10.3389/fendo.2025.1654129 | No cognitive outcomes: not reported |
| 2025 | Effects of acute aerobic and resistance exercise execution order on glycemia, blood pressure, and heart rate variability in postmenopausal women with type 2 diabetes mellitus: A randomized controlled trial | https://doi.org/10.1016/j.jbmt.2025.05.009 | No cognitive outcomes: not reported |
| 2022 | Exercise capacity is related to attenuated responses in oxygen extraction and left ventricular longitudinal strain in asymptomatic type 2 diabetes patients | https://doi.org/10.1093/eurjpc/zwaa007 | No cognitive outcomes: not reported |
| 2025 | Telemedicine-supported lifestyle intervention for glycemic control in patients with CHD and T2DM: multicenter, randomized controlled trial | https://doi.org/10.1038/s41591-025-03498-w | No cognitive outcomes: not reported |
| 2021 | Resistance Exercise Versus Aerobic Exercise Combined with Metformin Therapy in the Treatment of type 2 Diabetes: A 12-Week Comparative Clinical Study | https://doi.org/10.2174/1871530320999200918143227 | No cognitive outcomes: not reported |
| 2024 | Eight Weeks of Intermittent Exercise in Hypoxia, with or without a Low-Carbohydrate Diet, Improves Bone Mass and Functional and Physiological Capacity in Older Adults with Type 2 Diabetes | https://doi.org/10.3390/nu16111624 | No cognitive outcomes: not reported |
| 2024 | The effect of blood flow-restrictive resistance training on the risk of atherosclerotic cardiovascular disease in middle-aged patients with type 2 diabetes: a randomized controlled trial | https://doi.org/10.3389/fendo.2024.1482985 | No cognitive outcomes: not reported |
| 2025 | Impact of Exercise Manual Program on Biochemical Markers in Sedentary Prediabetic Patients: A Randomized Controlled Trial | https://doi.org/10.3390/medicina61020190 | No cognitive outcomes: not reported |
| 2024 | Increasing aerobic exercise intensity fails to consistently improve the glycemic response in people living with prediabetes or type 2 diabetes mellitus: the INTENSITY trial | https://doi.org/10.1139/apnm-2023-0495 | No cognitive outcomes: not reported |
| 2025 | The relationship between abdominal fat and sleep quality after combined exercise in patients with type 2 diabetes mellitus | https://doi.org/10.3389/fendo.2025.1471608 | No cognitive outcomes: not reported |
| 2022 | Impact of continuous vs. interval training on oxygen extraction and cardiac function during exercise in type 2 diabetes mellitus | https://doi.org/10.1007/s00421-022-04884-9 | No cognitive outcomes: not reported |
| 2013 | The effect of exercise training on ankle-brachial index in type 2 diabetes | https://doi.org/10.1016/j.atherosclerosis.2013.07.002 | No cognitive outcomes: not reported |
| 2019 | Effects of a 12-week moderate-intensity exercise training on blood glucose response in patients with type 2 diabetes: A prospective longitudinal study | https://doi.org/10.1097/MD.0000000000016860 | No cognitive outcomes: not reported |
| 2010 | Exercise ameliorates serum MMP-9 and TIMP-2 levels in patients with type 2 diabetes | https://doi.org/10.1016/j.diabet.2009.11.004 | No cognitive outcomes: not reported |
| 2025 | Psychosocial and physiological health outcomes of outdoor green exercise versus indoor exercise in knee osteoarthritis patients coexisting with type 2 diabetes mellitus: a randomized controlled trial | https://doi.org/10.3389/fendo.2025.1560536 | No cognitive outcomes: not reported |
| 2020 | Impact of combined training with different exercise intensities on inflammatory and lipid markers in type 2 diabetes: a secondary analysis from a 1-year randomized controlled trial | https://doi.org/10.1186/s12933-020-01136-y | No cognitive outcomes: not reported |
| 2021 | Effect of blood flow-restrictive resistance training on metabolic disorder and body composition in older adults with type 2 diabetes: a randomized controlled study | https://doi.org/10.3389/fendo.2024.1409267 | No cognitive outcomes: not reported |
| 2022 | A randomized controlled trial of Baduanjin exercise to reduce the risk of atherosclerotic cardiovascular disease in patients with prediabetes | https://doi.org/10.1038/s41598-022-22896-5 | No cognitive outcomes: not reported |
| 2014 | Exercise improves gait, reaction time and postural stability in older adults with type 2 diabetes and neuropathy | https://doi.org/10.1016/j.jdiacomp.2014.04.007 | No cognitive outcomes: not reported |
| 2022 | Effect of Exercise Training on Spexin Level, Appetite, Lipid Accumulation Product, Visceral Adiposity Index, and Body Composition in Adults With Type 2 Diabetes | https://doi.org/10.1177/10998004211050596 | No cognitive outcomes: not reported |
| 2022 | A Comparative Study of Health Efficacy Indicators in Subjects with T2DM Applying Power Cycling to 12 Weeks of Low-Volume High-Intensity Interval Training and Moderate-Intensity Continuous Training | https://doi.org/10.1155/2022/9273830 | No cognitive outcomes: not reported |
| 2021 | Effects of Acute Resistance Exercise with and without Whole-Body Electromyostimulation and Endurance Exercise on the Postprandial Glucose Regulation in Patients with Type 2 Diabetes Mellitus: A Randomized Crossover Study | https://doi.org/10.3390/nu13124322 | No cognitive outcomes: not reported |
| 2012 | Effect of exercise on blood pressure in type 2 diabetes: a randomized controlled trial | https://doi.org/10.1007/s11606-012-2103-8 | No cognitive outcomes: not reported |
| 2012 | Effects of exercise on sRAGE levels and cardiometabolic risk factors in patients with type 2 diabetes: a randomized controlled trial | https://doi.org/10.1210/jc.2012-1951 | No cognitive outcomes: not reported |
| 2009 | Effects of exercise intervention on myocardial function in type 2 diabetes | https://doi.org/10.1136/hrt.2009.165571 | No cognitive outcomes: not reported |
| 2010 | Postresistance exercise blood pressure reduction is influenced by exercise intensity in type-2 diabetic and nondiabetic individuals | https://doi.org/10.1519/JSC.0b013e3181d67488 | No cognitive outcomes: not reported |
| 2024 | Different intensities of aerobic training for patients with type 2 diabetes mellitus and knee osteoarthritis: a randomized controlled trial | https://doi.org/10.3389/fendo.2024.1463587 | No cognitive outcomes: not reported |
| 2010 | Aerobic exercise improves cognition for older adults with glucose intolerance, a risk factor for Alzheimer's disease | https://doi.org/10.3233/JAD-2010-100768 | No cognitive outcomes: not reported |
| 2018 | Response to a Supervised Structured Aerobic Exercise Training Program in Patients with Type 2 Diabetes Mellitus - Does Gender Make a Difference? A Randomized Controlled Clinical Trial | https://doi.org/10.1016/j.jnma.2017.10.003 | No cognitive outcomes: not reported |
| 2019 | Effects of Resistance Exercise on Glycated Hemoglobin and Functional Performance in Older Patients with Comorbid Diabetes Mellitus and Knee Osteoarthritis: A Randomized Trial | https://doi.org/10.3390/ijerph17010224 | No cognitive outcomes: not reported |
| 2016 | A randomized controlled trial on the effects of combined aerobic-resistance exercise on muscle strength and fatigue, glycemic control and health-related quality of life of type 2 diabetes patients | NA | No cognitive outcomes: not reported |
| 2013 | Exercise improves quality of life in indigenous Polynesian peoples with type 2 diabetes and visceral obesity | https://doi.org/10.1123/jpah.10.5.699 | No cognitive outcomes: not reported |
| 2012 | The impact of aerobic exercise training on novel adipokines, apelin and ghrelin, in patients with type 2 diabetes | https://doi.org/10.12659/msm.882734 | No cognitive outcomes: not reported |
| 2015 | An investigation and comparison of the effectiveness of different exercise programmes in improving glucose metabolism and pancreatic β cell function of type 2 diabetes patients | https://doi.org/10.1111/ijcp.12679 | No cognitive outcomes: not reported |
| 2015 | Supervised exercise training reduces oxidative stress and cardiometabolic risk in adults with type 2 diabetes: a randomized controlled trial | https://doi.org/10.1038/srep09238 | No cognitive outcomes: not reported |
| 2023 | Is isoenergetic high-intensity interval exercise superior to moderate-intensity continuous exercise for cardiometabolic risk factors in individuals with type 2 diabetes mellitus? A single-blinded randomized controlled study | https://doi.org/10.1080/17461391.2023.2167238 | No cognitive outcomes: not reported |
| 2015 | A 12-week sports-based exercise programme for inactive Indigenous Australian men improved clinical risk factors associated with type 2 diabetes mellitus | https://doi.org/10.1016/j.jsams.2014.06.013 | No cognitive outcomes: not reported |
| 2014 | The effects of aerobic exercise training at two different intensities in obesity and type 2 diabetes: implications for oxidative stress, low-grade inflammation and nitric oxide production | https://doi.org/10.1007/s00421-013-2769-6 | No cognitive outcomes: not reported |
| 2010 | Exercise prescription and the patient with type 2 diabetes: a clinical approach to optimizing patient outcomes | https://doi.org/10.1111/j.1745-7599.2010.00490.x | Non-randomized controlled trial design |
| 2023 | Diet, exercise, and pharmacotherapy for sarcopenia in people with diabetes | https://doi.org/10.1016/j.metabol.2023.155585 | Non-randomized controlled trial design |
| 2022 | MicroRNAs as biomarkers for monitoring cardiovascular changes in Type II Diabetes Mellitus (T2DM) and exercise | https://doi.org/10.1007/s40200-022-01066-4 | Non-randomized controlled trial design |
| 2022 | Ketogenic Diet Benefits to Weight Loss, Glycemic Control, and Lipid Profiles in Overweight Patients with Type 2 Diabetes Mellitus: A Meta-Analysis of Randomized Controlled Trails | https://doi.org/10.3390/ijerph191610429 | Non-randomized controlled trial design |
| 2022 | Effect of resistance training on HbA1c in adults with type 2 diabetes mellitus and the moderating effect of changes in muscular strength: a systematic review and meta-analysis | https://doi.org/10.1136/bmjdrc-2021-002595 | Non-randomized controlled trial design |
| 2019 | Obesity, risk of diabetes and role of physical activity, exercise training and cardiorespiratory fitness | https://doi.org/10.1016/j.pcad.2019.08.004 | Non-randomized controlled trial design |
| 2023 | Physical Exercise Methods and Their Effects on Glycemic Control and Body Composition in Adults with Type 2 Diabetes Mellitus (T2DM): A Systematic Review | https://doi.org/10.3390/ejihpe13110176 | Non-randomized controlled trial design |
| 2024 | Meta-analysis of the effect of exercise intervention on cognitive function in elderly patients with type 2 diabetes mellitus | https://doi.org/10.1186/s12877-024-05352-z | Non-randomized controlled trial design |
| 2024 | The Impact of Resistance Exercise Training on Glycemic Control Among Adults with Type 2 Diabetes: A Systematic Review and Meta-Analysis of Randomized Controlled Trials | https://doi.org/10.1177/10998004241246272 | Non-randomized controlled trial design |
| 2016 | Exercise for the diabetic brain: how physical training may help prevent dementia and Alzheimer's disease in T2DM patients | https://doi.org/10.1007/s12020-016-0976-8 | Non-randomized controlled trial design |
| 2017 | Effects of Exercise on Type 2 Diabetes Mellitus-Related Cognitive Impairment and Dementia | https://doi.org/10.3233/JAD-161154 | Non-randomized controlled trial design |
| 2021 | Exercise-Linked Irisin: Consequences on Mental and Cardiovascular Health in Type 2 Diabetes | https://doi.org/10.3390/ijms22042199 | Non-randomized controlled trial design |
| 2024 | Aerobic Exercise Improves the Overall Outcome of Type 2 Diabetes Mellitus Among People With Mental Disorders | https://doi.org/10.1155/da/6651804 | Non-randomized controlled trial design |
| 2025 | Impact of resistance training on cardiometabolic health-related indices in patients with type 2 diabetes and overweight/obesity: a systematic review and meta-analysis of randomised controlled trials | https://doi.org/10.1136/bjsports-2024-108947 | Non-randomized controlled trial design |
| 2021 | Exercise as a drug for glucose management and prevention in type 2 diabetes mellitus | https://doi.org/10.1016/j.coph.2021.05.006 | Non-randomized controlled trial design |
| 2019 | Physical Exercise as Therapy for Type 2 Diabetes Mellitus: From Mechanism to Orientation | https://doi.org/10.1159/000500110 | Non-randomized controlled trial design |
| 2018 | Exaggerated exercise pressor reflex in type 2 diabetes: Potential role of oxidative stress | https://doi.org/10.1016/j.autneu.2019.102591 | Non-randomized controlled trial design |
| 2016 | Exercise for the management of type 2 diabetes mellitus: factors to consider with current guidelines | https://doi.org/10.23736/S0022-4707.17.06969-9 | Non-randomized controlled trial design |
| 2019 | Exercise and Cardiovascular Risk among Masters Athletes with Type 2 Diabetes | https://doi.org/10.1007/s11892-019-1229-z | Non-randomized controlled trial design |
| 2019 | Relationship between diet/exercise and pharmacotherapy to enhance the GLP-1 levels in type 2 diabetes | https://doi.org/10.1002/edm2.68 | Non-randomized controlled trial design |
| 2025 | Tailoring Exercise Prescription for Effective Diabetes Glucose Management | https://doi.org/10.1210/clinem/dgae908 | Non-randomized controlled trial design |
| 2022 | Exercise and Nutrition Strategies for Combating Sarcopenia and Type 2 Diabetes Mellitus in Older Adults | https://doi.org/10.3390/jfmk7020048 | Non-randomized controlled trial design |
| 2015 | Chronotropic Incompetence During Exercise in Type 2 Diabetes: Aetiology, Assessment Methodology, Prognostic Impact and Therapy | https://doi.org/10.1007/s40279-015-0328-5 | Non-randomized controlled trial design |
| 2023 | Effect of Eccentric Exercise on Metabolic Health in Diabetes and Obesity | https://doi.org/10.1186/s40798-023-00596-2 | Non-randomized controlled trial design |
| 2021 | Effect of High-Intensity Interval Training on Quality of Life, Sleep Quality, Exercise Motivation and Enjoyment in Sedentary People with Type 1 Diabetes Mellitus | https://doi.org/10.3390/ijerph182312612 | Ineligible participant characteristics |
| 2005 | Exercise with and without an insulin pump among children and adolescents with type 1 diabetes mellitus | https://doi.org/10.1542/peds.2004-2428 | Ineligible participant characteristics |
| 2018 | Glycemic, inflammatory and oxidative stress responses to different high-intensity training protocols in type 1 diabetes: A randomized clinical trial | https://doi.org/10.1016/j.jdiacomp.2018.09.008 | Ineligible participant characteristics |
| 2018 | LY3298176, a novel dual GIP and GLP-1 receptor agonist for the treatment of type 2 diabetes mellitus: From discovery to clinical proof of concept | https://doi.org/10.1016/j.molmet.2018.09.009 | Ineligible participant characteristics |
| 2025 | Effect of rhPTH(1-34) and alendronate on the treatment of type 2 diabetic bone disease | https://doi.org/10.3389/fendo.2025.1657481 | Ineligible participant characteristics |
| 2019 | PDE5 Inhibition Stimulates Tie2-Expressing Monocytes and Angiopoietin-1 Restoring Angiogenic Homeostasis in Diabetes | https://doi.org/10.1210/jc.2018-02525 | Ineligible participant characteristics |
| 2020 | Endogenously released GIP reduces and GLP-1 increases hepatic insulin extraction | https://doi.org/10.1016/j.peptides.2019.170231 | Ineligible participant characteristics |
| 2013 | Effects of LX4211, a dual SGLT1/SGLT2 inhibitor, plus sitagliptin on postprandial active GLP-1 and glycemic control in type 2 diabetes | https://doi.org/10.1016/j.clinthera.2013.01.010 | Ineligible participant characteristics |
| 2022 | The benefit of exercise rehabilitation guided by 6-minute walk test on lipoprotein-associated phospholipase A2 in patients with coronary heart disease undergoing percutaneous coronary intervention: a prospective randomized controlled study | https://doi.org/10.1186/s12872-021-02430-7 | Ineligible participant characteristics |
| 2021 | Adiponectin in relation to exercise and physical performance in patients with type 2 diabetes and coronary artery disease | https://doi.org/10.1080/21623945.2021.1996699 | Ineligible participant characteristics |
| 2020 | Effects of sitagliptin on exercise capacity and hemodynamics in patients with type 2 diabetes mellitus and coronary artery disease | https://doi.org/10.1007/s00380-019-01526-7 | Ineligible participant characteristics |
| 2024 | Carnosine supplementation improves glucose control in adults with pre-diabetes and type 2 diabetes: A randomised controlled trial | https://doi.org/10.1016/j.numecd.2023.10.012 | Ineligible participant characteristics |
| 2011 | The effect of acute exercise on undercarboxylated osteocalcin in obese men | https://doi.org/10.1007/s00198-010-1370-7 | Ineligible participant characteristics |
| 2012 | Effect of 12-week resistance exercise program on body composition, muscle strength, physical function, and glucose metabolism in healthy, insulin-resistant, and diabetic elderly Icelanders | https://doi.org/10.1093/gerona/gls096 | Ineligible participant characteristics |
| 2023 | Effect of resistance vs. aerobic exercise in pre-diabetes: an RCT | https://doi.org/10.1186/s13063-023-07116-3 | Ineligible participant characteristics |
| 2020 | Adiposity measures and pre-diabetes or diabetes in adults with hypertension in Singapore polyclinics | https://doi.org/10.1111/jch.13587 | Ineligible participant characteristics |
| 2024 | Exploring the Effect of Exercise versus Metformin on Insulin Resistance amongst Nigerians with Pre-diabetes: A Randomised Controlled Trial | https://doi.org/10.4103/npmj.npmj_148_24 | Ineligible participant characteristics |
| 2023 | Automated Insulin Delivery in Women with Pregnancy Complicated by Type 1 Diabetes | https://doi.org/10.1056/NEJMoa2303911 | Ineligible participant characteristics |
| 2024 | Resistance exercise lowers blood pressure and improves vascular endothelial function in individuals with elevated blood pressure or stage-1 hypertension | https://doi.org/10.1152/ajpheart.00386.2023 | Ineligible participant characteristics |
| 2015 | Effects of the DASH Diet and Walking on Blood Pressure in Patients With Type 2 Diabetes and Uncontrolled Hypertension: A Randomized Controlled Trial | https://doi.org/10.1111/jch.12597 | Ineligible participant characteristics |
| 2017 | A randomized clinical trial of exercise during pregnancy to prevent gestational diabetes mellitus and improve pregnancy outcome in overweight and obese pregnant women | https://doi.org/10.1016/j.ajog.2017.01.037 | Ineligible participant characteristics |
| 2009 | Short-term aerobic exercise reduces arterial stiffness in older adults with type 2 diabetes, hypertension, and hypercholesterolemia | https://doi.org/10.2337/dc09-0149 | Ineligible participant characteristics |
| 2023 | The Effect of a mHealth App (KENPO-app) for Specific Health Guidance on Weight Changes in Adults With Obesity and Hypertension: Pilot Randomized Controlled Trial | https://doi.org/10.2196/43236 | Ineligible participant characteristics |
| 2018 | Exercise in patients with hypertension and chronic kidney disease: a randomized controlled trial | https://doi.org/10.1038/s41371-018-0055-0 | Ineligible participant characteristics |
| 2022 | Exercise training and high-sensitivity cardiac troponin-I in patients with heart failure with reduced ejection fraction | https://doi.org/10.1002/ehf2.14674 | Ineligible participant characteristics |
| 2010 | Aerobic training restores arterial baroreflex sensitivity in older adults with type 2 diabetes, hypertension, and hypercholesterolemia | https://doi.org/10.1097/JSM.0b013e3181ea8454 | Ineligible participant characteristics |
| 2021 | FEASibility testing a randomized controlled trial of an exercise program to improve cognition for T2DM patients (the FEAST trial): A study protocol | https://doi.org/10.1002/nur.22174 | Unable to access the full -text |
| 2014 | Effects of vildagliptin compared with glibenclamide on glucose variability after a submaximal exercise test in patients with type 2 diabetes: study protocol for a randomized controlled trial, DIABEX VILDA | https://doi.org/10.1186/1745-6215-15-424 | Unable to access the full -text |
| 2018 | Study protocol: a randomised controlled trial of supervised resistance training versus aerobic training in Sri Lankan adults with type 2 diabetes mellitus: SL-DART study | https://doi.org/10.1186/s12889-018-5069-6 | Unable to access the full -text |
| 2012 | A randomised trial comparing low-fat diets differing in carbohydrate and protein ratio, combined with regular moderate intensity exercise, on glycaemic control, cardiometabolic risk factors, food cravings, cognitive function and psychological wellbeing in adults with type 2 diabetes: Study protocol | https://doi.org/10.1016/j.cct.2015.11.001 | Unable to access the full -text |
| 2020 | Effectiveness of digital health using the transtheoretical model to prevent or delay type 2 diabetes in impaired glucose tolerance patients: protocol for a randomized control trial | https://doi.org/10.1186/s12889-019-7921-8 | Unable to access the full -text |
| 2019 | Smart Phone APP to Restore Optimal Weight (SPAROW): protocol for a randomised controlled trial for women with recent gestational diabetes | https://doi.org/10.1186/s12889-019-7691-3 | Unable to access the full -text |
| 2020 | Long-term Change in Physiological Markers and Cognitive Performance in Type 2 Diabetes: The Look AHEAD Study | https://doi.org/10.1210/clinem/dgaa591 | Unable to access the full -text |
| 2009 | Relationship between baseline glycemic control and cognitive function in individuals with type 2 diabetes and other cardiovascular risk factors: the action to control cardiovascular risk in diabetes-memory in diabetes (ACCORD-MIND) trial | https://doi.org/10.2337/dc08-1153 | Unable to access the full -text |
| 2020 | Long Term Dietary Restriction of Advanced Glycation End-Products (AGEs) in Older Adults with Type 2 Diabetes Is Feasible and Efficacious-Results from a Pilot RCT | https://doi.org/10.3390/nu12103143 | Unable to access the full -text |
| 2020 | Long-term Change in Physiological Markers and Cognitive Performance in Type 2 Diabetes: The Look AHEAD Study | https://doi.org/10.1210/clinem/dgaa591 | Unable to access the full -text |
| 2018 | Sex-related differences in the prevalence of cognitive impairment among overweight and obese adults with type 2 diabetes | https://doi.org/10.1016/j.jalz.2018.05.015 | Unable to access the full -text |
| 2012 | Effect of communicating genetic and phenotypic risk for type 2 diabetes in combination with lifestyle advice on objectively measured physical activity: protocol of a randomised controlled trial | https://doi.org/10.1186/1471-2458-12-444 | Unable to access the full -text |
| 2014 | A low glycaemic load breakfast can attenuate cognitive impairments observed in middle aged obese females with impaired glucose tolerance | https://doi.org/10.1016/j.numecd.2014.04.015 | Unable to access the full -text |
| 2019 | Hypoglycemia and Incident Cognitive Dysfunction: A Post Hoc Analysis From the ORIGIN Trial | https://doi.org/10.2337/dc18-0690 | Unable to access the full -text |
| 2018 | Results From a Feasibility Study of Square-Stepping Exercise in Older Adults With Type 2 Diabetes and Self-Reported Cognitive Complaints to Improve Global Cognitive Functioning | https://doi.org/10.1016/j.jcjd.2018.02.003 | Unable to access the full -text |
| 2023 | Effects of high-intensity interval training, moderate-intensity continuous training, and guideline-based physical activity on cardiovascular metabolic markers, cognitive and motor function in elderly sedentary patients with type 2 diabetes (HIIT-DM): a protocol for a randomized controlled trial | https://doi.org/10.3389/fnagi.2023.1211990 | Unable to access the full -text |
| 2018 | Sleep duration, obesity and insulin resistance in a multi-ethnic UK population at high risk of diabetes | https://doi.org/10.1016/j.diabres.2018.03.010 | No appropriate control group |
| 2023 | Psychological resilience in older adults with type 2 diabetes from the Look AHEAD Trial | https://doi.org/10.1111/jgs.17986 | No appropriate control group |
| 2022 | Factors associated with patient empowerment in Spanish adults with type 2 diabetes: A cross-sectional analysis | https://doi.org/10.1111/hex.13501 | No appropriate control group |
| 2023 | Diagnosis of coronary artery disease in patients with type 2 diabetes mellitus based on computed tomography and pericoronary adipose tissue radiomics: a retrospective cross-sectional study | https://doi.org/10.1186/s12933-023-01748-0 | No appropriate control group |
| 2015 | The relationship between anthropometric indices and type 2 diabetes mellitus among adults in north-east China | https://doi.org/10.1017/S1368980014002250 | No appropriate control group |
| 2019 | Aerobic Fitness and Adherence to Guideline-Recommended Minimum Physical Activity Among Ambulatory Patients With Type 2 Diabetes Mellitus | https://doi.org/10.2337/dc18-2634 | No appropriate control group |
| 2018 | Sedentary Time and MRI-Derived Measures of Adiposity in Active Versus Inactive Individuals | https://doi.org/10.1002/oby.22034 | No appropriate control group |
| 2017 | Type 2 Diabetes Mellitus Is Associated With Better Bone Microarchitecture But Lower Bone Material Strength and Poorer Physical Function in Elderly Women: A Population-Based Study | https://doi.org/10.1002/jbmr.3057 | No appropriate control group |
| 2006 | Recruitment of Older Veterans with Diabetes Risk for Alzheimer's Disease for a Randomized Clinical Trial of Computerized Cognitive Training | https://doi.org/10.3233/JAD-180952 | No appropriate control group |
| 2025 | Td2Ast project: A pragmatic intervention on diet and physical activity for patients with type 2 diabetes mellitus | https://doi.org/10.1016/j.pcd.2025.02.003 | No appropriate control group |
| 2010 | Long- but not short-term multifactorial intervention with focus on exercise training improves coronary endothelial dysfunction in diabetes mellitus type 2 and coronary artery disease | https://doi.org/10.1093/eurheartj/ehp398 | No appropriate control group |

**Table S3** Abbreviations and definitions of intervention measures

| Intervention | Abbreviation | Definition |
| --- | --- | --- |
| Multimodal exercise | ME | Resistance training + balance training, aerobic exercise + flexibility training, etc |
| Aerobic exercise | AE | Walking, jogging, swimming, cycling, etc |
| Resistance exercise | RE | Dumbbell lifting, resistance band training, squats, push-ups, etc |
| Mind–body exercise | MBE | Tai chi, yoga, etc |
| Usual care | Usual | There are no specific interventions |

**Figure S1** Ranking probability plots illustrating the posterior likelihood of each intervention achieving the highest rank for global cognitive function improvement.


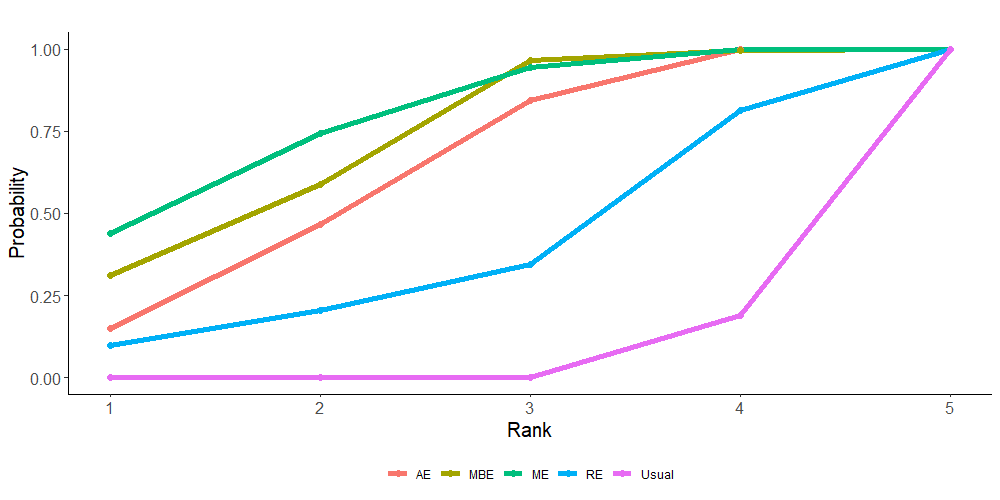


**Figure S2** Forest plot of the network meta-analysis comparing interventions for cognitive function assessed by the MoCA scale.


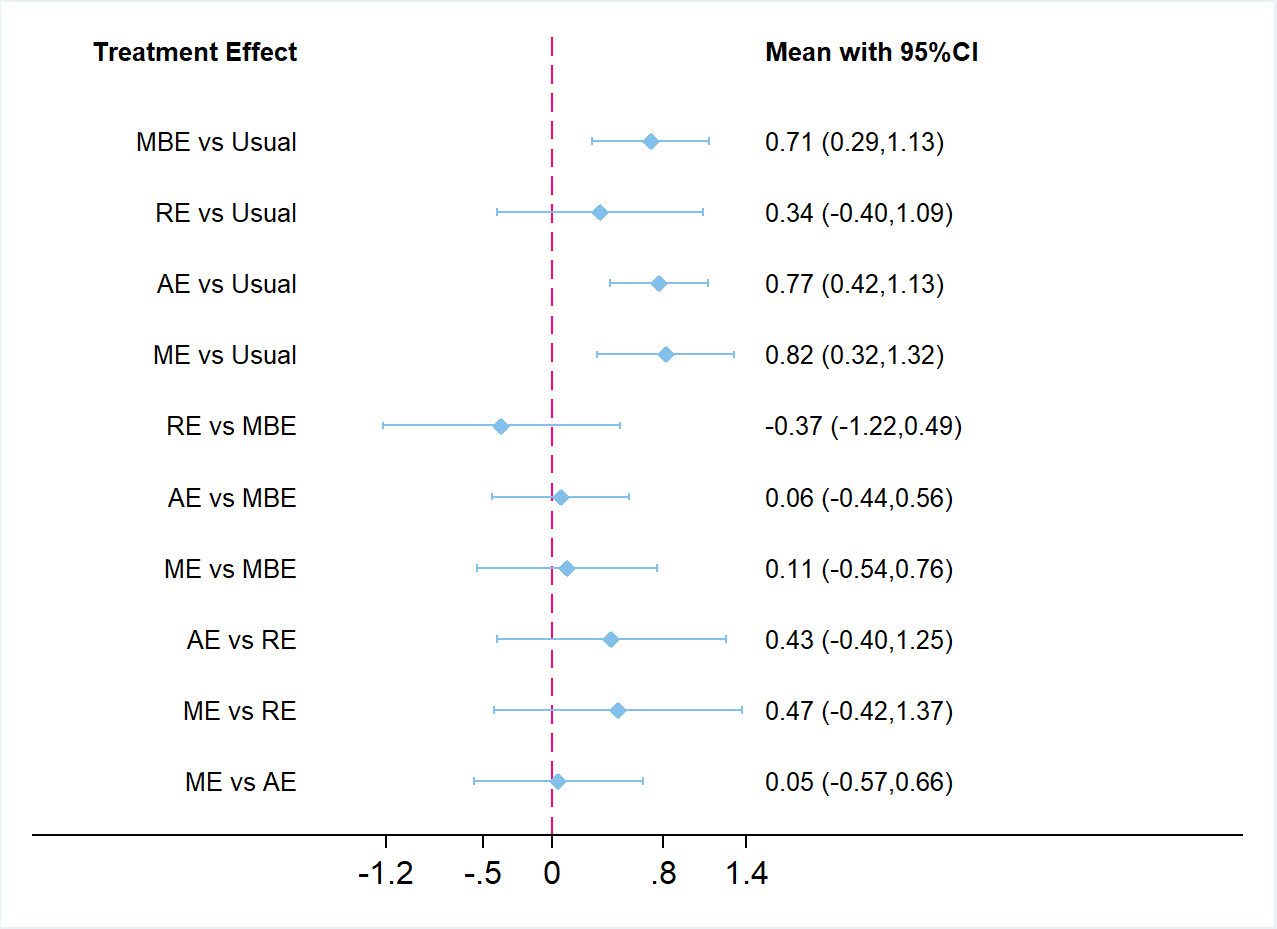


Effect sizes are presented as standardized mean differences (SMD) with 95% confidence intervals (CI).

**Table S4** League table from the network meta-analysis of interventions under the MoCA scale.

| ME |  |  |  |  |
| --- | --- | --- | --- | --- |
| 0.05(-0.57,0.66) | AE |  |  |  |
| 0.47(-0.42,1.37) | 0.43(-0.40,1.25) | RE |  |  |
| 0.11(-0.54,0.76) | 0.06(-0.44,0.56) | -0.37(-1.22,0.49) | MBE |  |
| **0.71(0.29,1.13)** | **0.77(0.42,1.13)** | 0.34(-0.40,1.09) | **0.71(0.29,1.13)** | Usual |

**Figure S3** Forest plot of the network meta-analysis comparing interventions for cognitive function assessed by the MMSE scale.


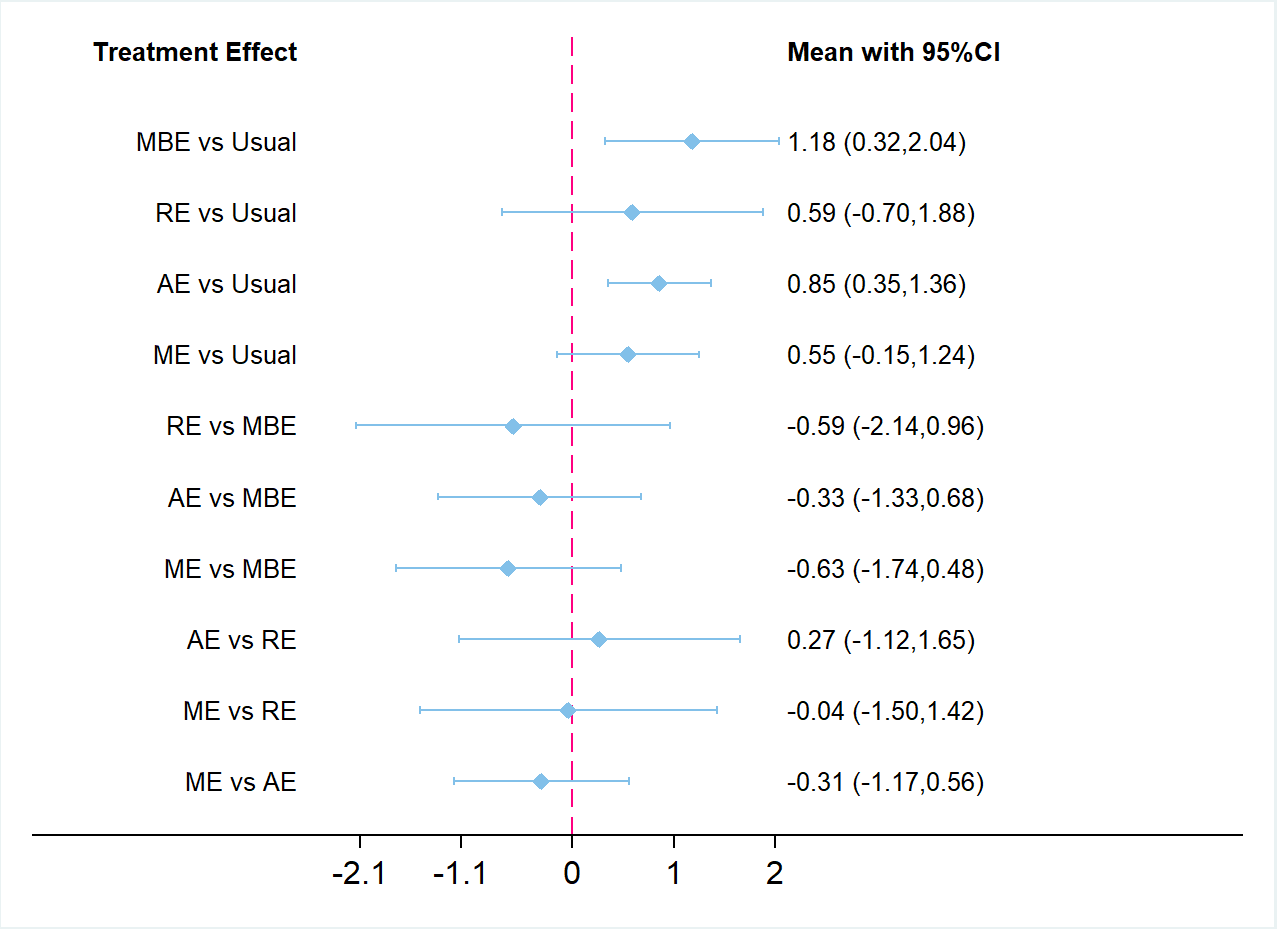


Effect sizes are presented as standardized mean differences (SMD) with 95% confidence intervals (CI).

**Table S5** League table from the network meta-analysis of interventions under the MMSE scale

| ME |  |  |  |  |
| --- | --- | --- | --- | --- |
| -0.31(-1.17,0.56) | AE |  |  |  |
| -0.04(-1.50,1.42) | 0.27 (-1.12,1.65) | RE |  |  |
| -0.63(-1.74,0.48) | -0.33(-1.33,0.68) | -0.59(-2.14,0. 69) | MBE |  |
| 0.55(-0.15,1.24) | **0.85(0.35,1. 36)** | 0.59(-0.70,1.88) | **1.18(0.32,2.04)** | Usual |

**Figure S4** Forest plot of interventions for cognitive function with a duration of ≤3 months.


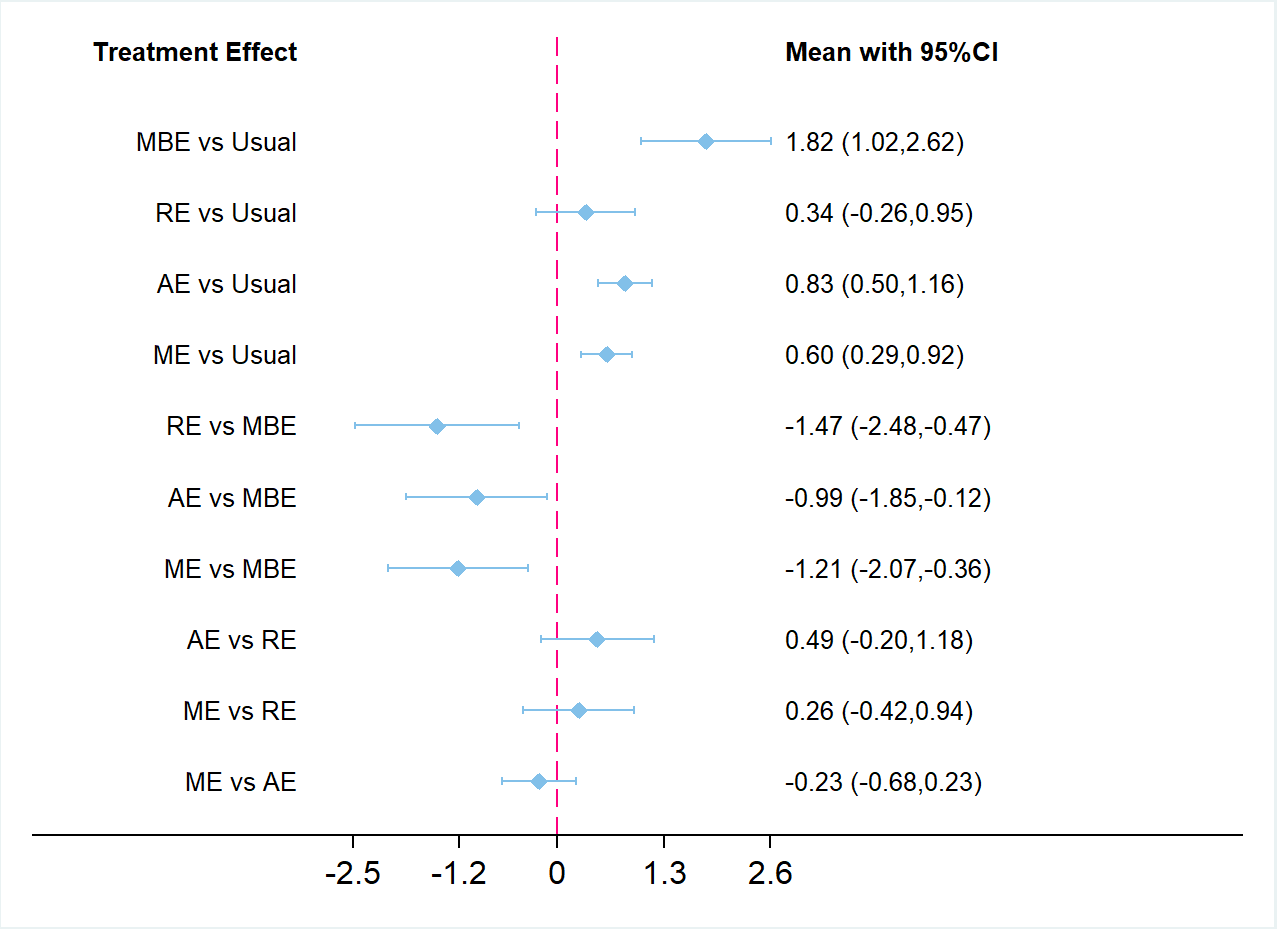


Effect sizes are presented as standardized mean differences (SMD) with 95% confidence intervals (CI).

**Table S6** League table of the network meta-analysis for cognitive function with intervention durations of ≤3 months.

| ME |  |  |  |  |
| --- | --- | --- | --- | --- |
| -0.23(-0.68,0.23) | AE |  |  |  |
| 0.26(-0.42,0.94) | 0.49 (-0.20,1.18) | RE |  |  |
| **-1.21(-2.07,-0.36)** | **-0.99(-1.85,-0.12)** | **-1.47 (-2.48,-0.47)** | MBE |  |
| **0.60(0.29,0.92)** | **0.83(0. 50,1. 16)** | 0.34(-0.26,0.95) | **1.82(1.02,2.62)** | Usual |

**Figure S5** Forest plot of interventions for cognitive function with a duration of >3 months.


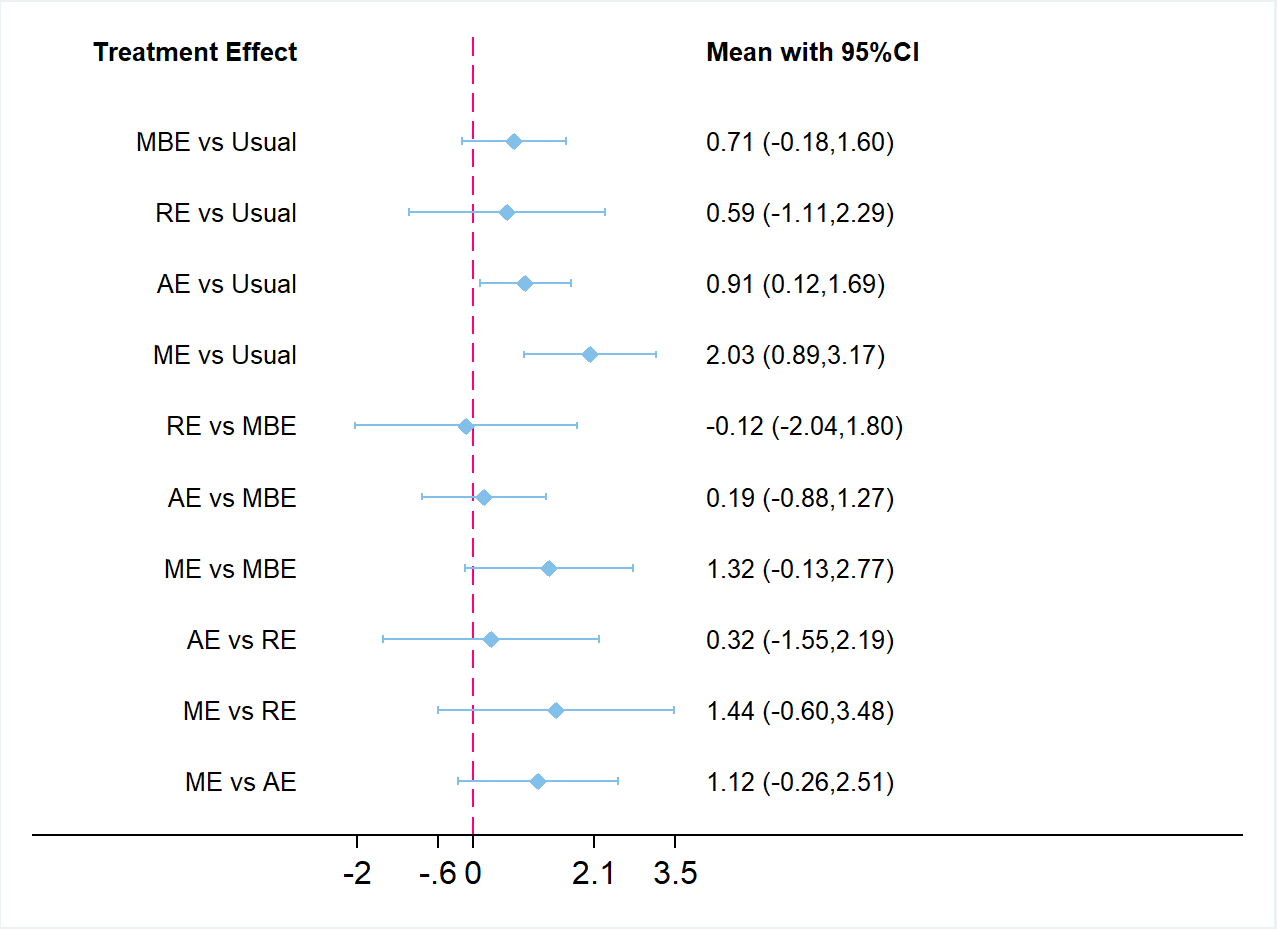


Effect sizes are presented as standardized mean differences (SMD) with 95% confidence intervals (CI).

**Table S7**League table of the network meta-analysis for cognitive function with intervention durations of >3 months.

| ME |  |  |  |  |
| --- | --- | --- | --- | --- |
| 1.12(-0.26,2.51) | AE |  |  |  |
| 1.44(-0.60,3.48) | 0.32 (-1.55,2.19) | RE |  |  |
| 1.32(-0.13,2.77) | 0.19 (-0.88,-1.27) | -0.12 (-2.04,1.80) | MBE |  |
| **2.03(0.89,3.17)** | **0.91(0. 12,1. 69)** | 0.59(-1.11,2.29) | 0.71(-0.18,1.60) | Usual |

**Figure S6** Forest plot of interventions for cognitive function with a training frequency of ≤3 sessions per week.


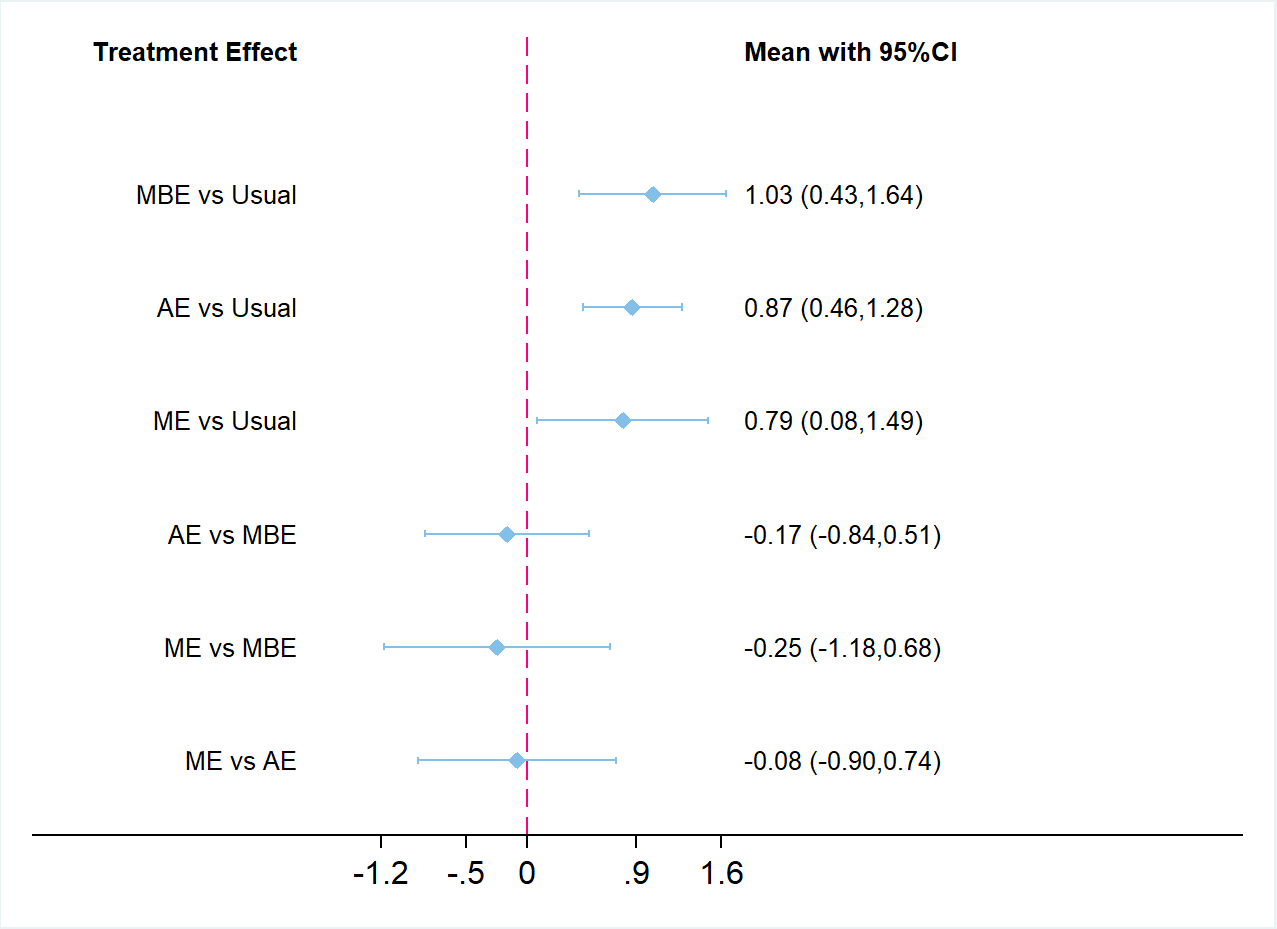


Effect sizes are presented as standardized mean differences (SMD) with 95% confidence intervals (CI).

**Table S8** League table of the network meta-analysis for cognitive function with training frequencies of ≤3 sessions per week.

| ME |  |  |  |
| --- | --- | --- | --- |
| -0.08 (-0.90,0.74) | AE |  |  |
| -0.25 (-1.18,0.68) | -0.17 (-0.84,0.51) | MBE |  |
| **0.79(0.08,1.49)** | 0.87(0.46,1.28) | 1.03(0.43,1.64) | Usual |

**Figure S7** Forest plot of interventions for cognitive function with a training frequency of >3 sessions per week.


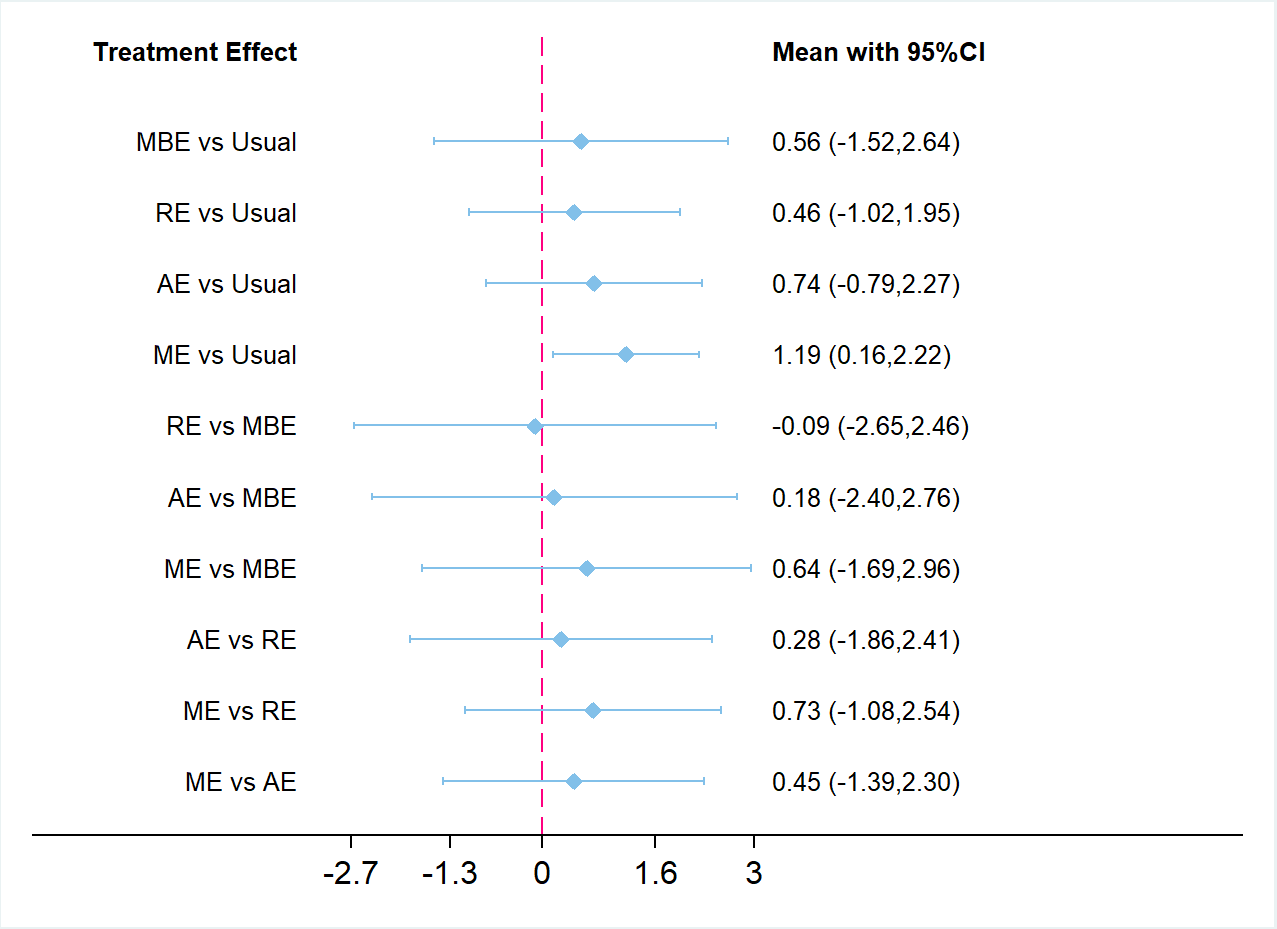


Effect sizes are presented as standardized mean differences (SMD) with 95% confidence intervals (CI).

**Table S9** League table of the network meta-analysis for cognitive function with training frequencies of >3 sessions per week

| ME |  |  |  |  |
| --- | --- | --- | --- | --- |
| 0.45(-1.39,2.30) | AE |  |  |  |
| 0.73(-1.08,2.54) | 0.28 (-1.86,2.41) | RE |  |  |
| 0.64(-1.69,2.96) | 0.18 (-2.40,2.76) | -0.09 (-2.65,2.46) | MBE |  |
| **1.19(0.16,2.22)** | 0.74(-0.79,2.27) | 0.46(-1.02,1.95) | 0.56(-1.52,2.64) | Usual |

**Figure S8** Forest plot of interventions for cognitive function in the sensitivity analysis.


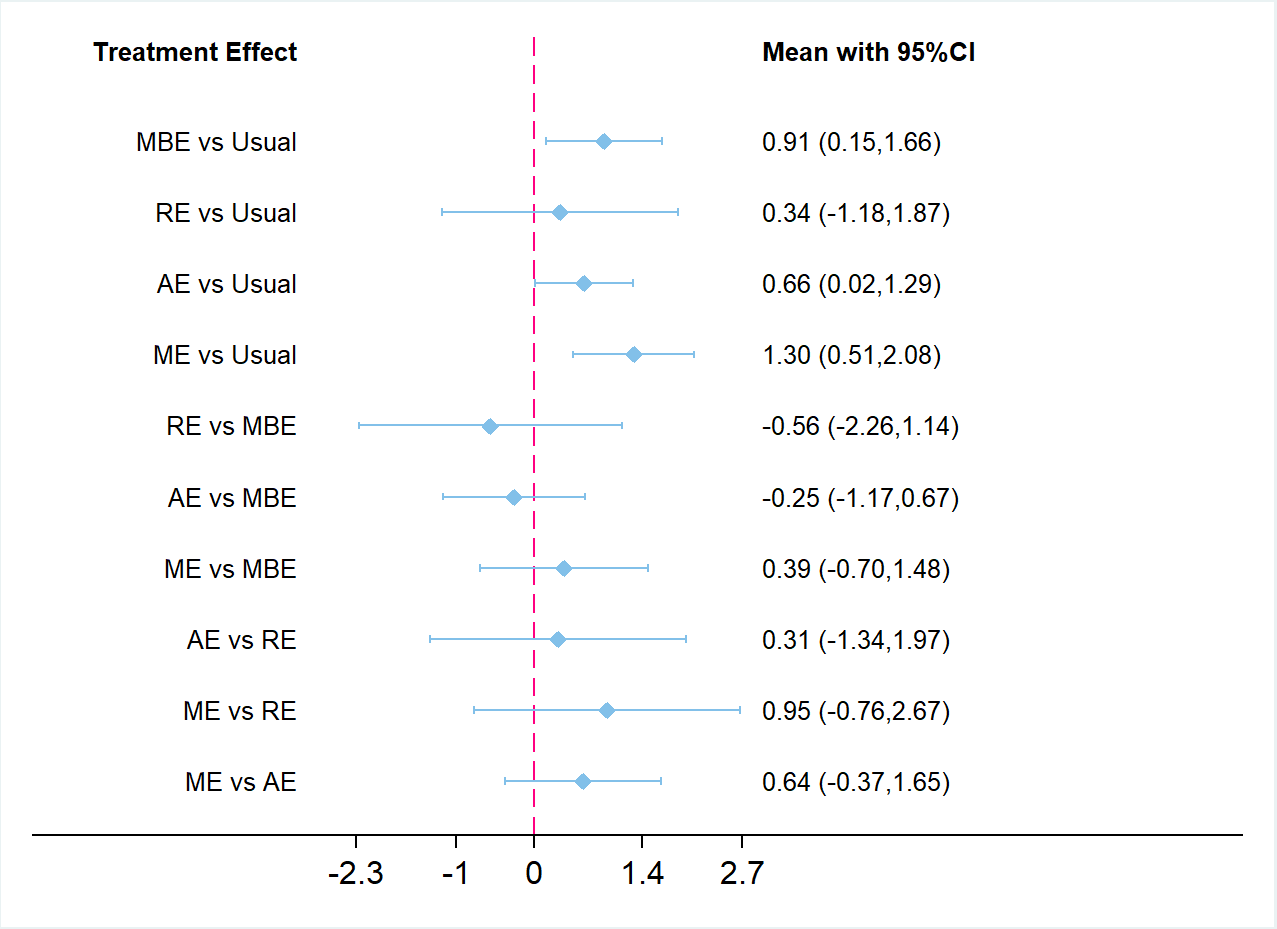


Effect sizes are presented as standardized mean differences (SMD) with 95% confidence intervals (CI).

**Table S10** League table of the network meta-analysis for cognitive function in the sensitivity analysis

| ME |  |  |  |  |
| --- | --- | --- | --- | --- |
| 0.64(-0.37,1.65) | AE |  |  |  |
| 0.95(-1.34,1.97) | 0.31(-1.34,1.97) | RE |  |  |
| 0.39(-0.70,1.48) | -0.25(-1.17,0.67) | -0.56(-2.26,1.14) | MBE |  |
| **1.30 (0.51,2.08)** | **0.66(0.02,1.29)** | 0.34(-1.18,1.87) | **0.91(0.15,1.66)** | Usual |
